# Building nonenhanced CT based radiomics model in discriminating arteriovenous malformation related hematomas from hypertensive intracerebral hematomas

**DOI:** 10.3389/fnins.2023.1284560

**Published:** 2023-11-28

**Authors:** Huanhuan Xie, Fei Dong, Ruiting Zhang, Xinfeng Yu, Peng Xu, Yinshan Tang, Peiyu Huang, Chao Wang

**Affiliations:** ^1^Department of Radiology, The Second Affiliated Hospital, Zhejiang University School of Medicine, Hangzhou, China; ^2^Neuroscience Intensive Care Unit, The Second Affiliated Hospital, Zhejiang University School of Medicine, Hangzhou, China; ^3^Department of Rehabilitation in Traditional Chinese Medicine, The Second Affiliated Hospital of Zhejiang University School of Medicine, Hangzhou, China

**Keywords:** hematoma, arteriovenous malformations, hypertensive intracerebral hematomas, CT, radiomics

## Abstract

**Objective:**

To develop and validate radiomics models on non-enhanced CT for discrimination of arteriovenous malformation (AVM) related hematomas from hypertensive intracerebral hematomas.

**Materials and methods:**

A total of 571 patients with acute intraparenchymal hematomas and baseline non-enhanced CT scans were retrospectively analyzed, including 297 cases of AVM related hematomas and 274 cases of hypertensive intracerebral hematomas. The patients were divided into training and validation cohorts in a 7:3 ratio with a random seed. A total of 1,688 radiomics features of hematomas were extracted from non-enhanced CT. Then, the least absolute shrinkage and selection operator (LASSO) regression was applied to select features and construct the radiomics models. In this study, a radiomics-based model was constructed that based on the radiomics features only. Furthermore, a combined model was constructed using radiomics features, clinical characteristics and radiological signs by radiologists’ evaluation. In addition, we compared predictive performance of the two models for discrimination of AVM related hematomas from hypertensive intracerebral hematomas.

**Results:**

A total of 67 radiomics features were selected to establish radiomics signature via LASSO regression. The radiomics-based model was constructed with 2 classifiers, support vector machine (SVM) and logistic regression (LR). AUCs of the radiomics-based model in the training set were 0.894 and 0.904, in validation set were 0.774 and 0.782 in SVM classifier and LR classifier, respectively. AUCs of the combined model (combined with radiomics, age and calcification) in the training set were 0.976 and 0.981, in validation set were 0.896 and 0.907 in SVM classifier and LR classifier, respectively. The combined model showed greater AUCs than radiomics-based model in both training set and validation set.

**Conclusion:**

The combined model using radiomics, age and calcification showed a satisfactory predictive performance for discrimination of AVM related hematomas from hypertensive intracerebral hematomas and hold great potential for personalized clinical decision.

## Introduction

Intracerebral hemorrhage is the most severe form of stroke due to its high rate of mortality exceeding 50% (Brouwers et al., 2014; Poon et al., 2014). Non-enhanced CT scan is the preferred initial imaging modality for patients presenting to the emergency department with suspected acute intracerebral hemorrhage (Heit et al., 2017). The most common cause of spontaneous intraparenchymal hematomas is hypertension (Balami and Buchan, 2012). However, early discrimination of another common cause, arteriovenous malformations (AVM) (Fukuda et al., 2017), is of more clinical significance, since excision or embolization of the ruptured AVM nidus is necessary to prevent re-hemorrhage. Currently, discriminating AVM related hematomas from hypertensive intracerebral hematomas on nonenhanced CT is challenging for the naked eye by radiologists. Angiography is the gold standard for diagnosing AVM, including CT angiography (CTA) and digital subtraction angiography (DSA). However, compared to non-enhanced CT scans, angiography is a more time-consuming and invasive procedure that requires contrast injection and patient compliance.

Radiomics is a quantitative analysis that utilizes high-throughput methods to extract a vast array of imaging features from radiological medical images. Radiomics is widely employed in phenotypic subtype classification as well as prognostic predictions of solid tumors (Wang et al., 2019; Li et al., 2022). In recent years, non-enhanced CT based radiomics have also been utilized to predict hematoma expansion after intracerebral hemorrhage, and all models in these studies demonstrated excellent predictive performance (Xie et al., 2020; Song et al., 2021). However, no study has focused on building a non-enhanced CT based radiomics model to differentiate AVM related hematomas from hypertensive intracerebral hematomas. AVM related hematomas exhibit greater compositional heterogeneity due to the presence of malformed vasculature within the hematomas, and the hematoma may be surrounded by dilated veins that can cause border indentation. Therefore, we postulate that non-enhanced CT based radiomics, an emerging technique for analyzing the shape and texture features of lesions (Gillies et al., 2016), can achieve a good predictive performance in discriminating AVM related hematomas from hypertensive intracerebral hematomas. Therefore, the aim of this study was to construct and validate a radiomics prediction model based on non-enhanced CT images in discriminating AVM related hematomas from hypertensive intracerebral hematomas.

## Materials and methods

### Patients and data acquisition

The present study was approved by the Institutional Review Boards of the Second Affiliated Hospital of Zhejiang University School of Medicine, and the written informed consent was waived. We searched our retrospectively maintained database for consecutive patients with acute hypertensive and AVM related spontaneous intracerebral hemorrhage between June 2011 and December 2022. All patients received baseline non-enhanced CT scans. Patients with artifacts in their CT images or on anticoagulation/antiplatelet therapy were excluded. Other causes of bleeding, such as moyamoya disease, intracranial aneurysms, cerebral amyloid angiopathy, or neoplastic related bleeding have been excluded in all confirmed patients. In total, 274 cases of hypertensive intraparenchymal hematomas and 297 cases of AVM related hematomas were included. Baseline nonenhanced CT images were acquired after admission to our hospital with either Siemens or GE medical systems. The scanning energy was 120 or 140 KVP and smart mAs were used. Slice thickness was 5 mm and the pixel spacing was 0.45 × 0.45 mm^2^ or 0.49 × 0.49 mm^2^.

### Hematomas segmentation and radiomics feature extraction

The segmentation of hematomas was performed using 3D-Slicer software (version 4.13.0, www.slicer.org) by a radiologist with 5 years of experience in neuroimaging. Volume of interest (VOI) were semi-automatically delineated on each slice of the CT image containing the entire lesion. Then, the segmentations were validated by another radiologist with 10 years of experience in neuroimaging in a cohort of 30 randomly selected patients. Interobserver intraclass correlation coefficient (ICC) > 0.75 was deemed to have a good reliability or reproducibility (Gstoettner et al., 2007).

A total of 1,688 quantitative imaging features were extracted from CT images with Radcloud platform (Huiying Medical Technology Co., Ltd., http://radcloud.cn) (Nie et al., 2020, 2021). The 1,688 radiomics features were extracted from each original and filtered segmentation and divided into five groups: (Brouwers et al., 2014) intensity [histogram-derived first-order statistics (*n* = 18)]; (Poon et al., 2014) shape (*n* = 14); (Heit et al., 2017) textural matrix [i.e., the gray-level co-occurrence matrix (GLCM), Gray Level Dependence Matrix (GLDM), gray-level run length matrix (GLRLM), gray-level size-zone matrix (GLSZM), and the neighborhood gray-tone difference matrix (NGTDM), *n* = 75]; (Balami and Buchan, 2012) wavelet-based transform (*n* = 744), and (Fukuda et al., 2017) other transforms (*n* = 837). The details of the radiomics features are shown in Supplementary Table S1.

### Machine learning-based radiomics prediction model construction

The validation set and training set were separated by random method with ratio 3:7, and the random seeds is 314. The statistical analysis was performed in Radcloud platform. As described above, a large number of image features may be computed. However, all these extracted features may not be useful for a particular task. Therefore, dimensionality reduction and selection of task-specific features for best performance are necessary steps. To reduce the redundant features, the feature selection methods included the least absolute shrinkage and selection operator (LASSO) were used for this purpose. For LASSO model, L1 regularizer was used as the cost function, and the error value of cross validation is 5, and the maximum number of iterations is 1,000.

Based on the selected features, the radiomics-based models were constructed with 2 classifiers, support vector machine (SVM) and logistic regression (LR), and the validation method was used to improve the effectiveness of the model. A combined model was also constructed which combined with the radiomics features, clinical characteristics and radiological signs. To assess the predictive performance, the receiver operating characteristic (ROC) curve, namely, area under curve (AUC) was used both in training set and validation set, respectively. And four indicators including P [precision = true positives / (true positives+ false positives)], R [recall = true positives / (true positives+ false negatives)], f1-score [f1-score = P*R*2/ (P + R)], support (total number in test set) to evaluate the performance of classifier in this study.

## Results

**Figure 1:**
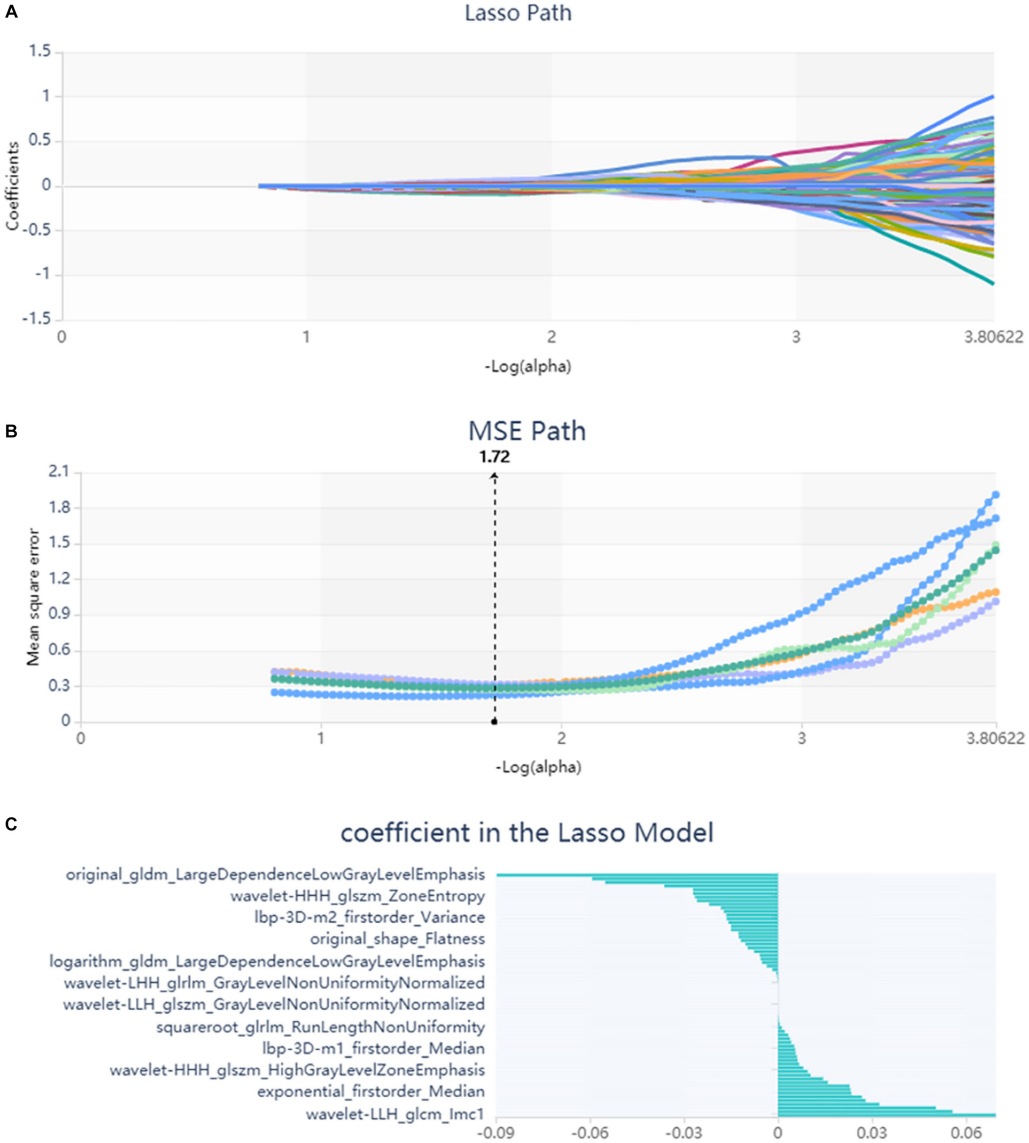
Lasso algorithm on feature select. **(A)** Lasso path; **(B)** MSE path; **(C)** coefficients in Lasso model. Using Lasso model, 67 features which are correspond to the optimal alpha value were selected.

**Figure 2:**
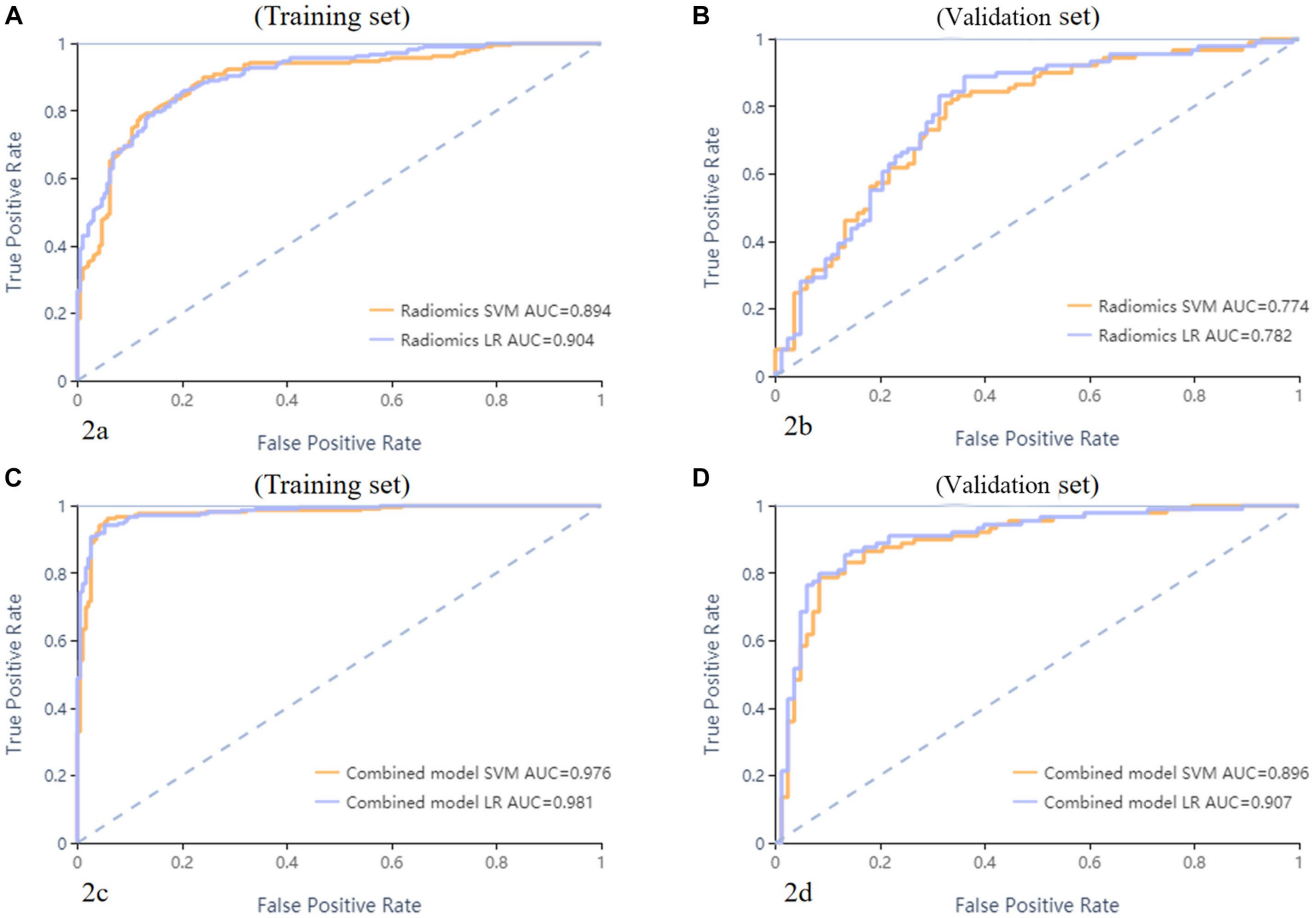
ROC curves of SVM and LR methods to classification. The radiomics based model ROC curve of training set, the AUCs of SVM and LR were 0.894 and 0.904, respectively **(A)**. The radiomics based model ROC curve of validate set, the AUCs of SVM and LR were 0.774 and 0.782, respectively **(B)**. The combined model ROC curve in training set, the AUCs of SVM and LR were 0.976 and 0.981, respectively **(C)**. The combined model ROC curve in validation set, the AUCs of SVM and LR were 0.896 and 0.907, respectively **(D)**.

A total of 571 patients (373 men and 198 women; mean age, 47.6 years ±18.97; range, 5–94 years) were included in this study. The training set comprised 265 males and 134 females (median age, 49 years; Range, 7–93 years), and the validation set included 108 males and 64 females (median age, 48.5 years; Range, 5–94 years). Both in the training and validation set, there were no significant differences in age and gender between training set and validation set. Age was significantly different between AVM related hematomas and hypertensive hematomas both in training set and validation set (*p* < 0.001), the sex did not differ significantly between the two groups. In the training and validation set, calcification was significantly different between AVM related hematomas and hypertensive hematomas (*p* < 0.001) (Table 1). Calcification was more commonly seen in AVM related hematomas than hypertensive hematomas (Table 1). A total of 67 features were selected from 1,688 features using LASSO method (Figure 1; Supplementary Table S2). Based on the selected features, there are several supervised learning classifiers available for classification analysis, which creates models that attempt to separate or predict the data with respect to an outcome or phenotype. In this study, the predicted models were constructed with SVM and LR classifiers, and the validation method was used to validate the effectiveness of the models. ROC curve analysis results were showed in Table 2 for training set and Table 3 for validation set. When training with SVM classifier, the AUC of radiomics-based model and combined model in training set were 0.894 (95% CI: 0.858–0.932; sensitivity = 0.83 and specificity = 0.82) and 0.976 (95% CI: 0.953–0.999; sensitivity = 0.95 and specificity = 0.95), and the AUC of validation set were 0.774 (95% CI: 0.705–0.843; sensitivity = 0.72 and specificity = 0.71) and 0.896 (95% CI: 0.841–0.951; sensitivity = 0.88 and specificity = 0.8), respectively (Figure 2). When training with LR classifier, the AUC of radiomics-based model and combined model in training set were 0.904 (95% CI, 0.866–0.942; sensitivity = 0.82 and specificity = 0.82) and 0.981 (95% CI, 0.958–1.000; sensitivity = 0.94 and specificity = 0.95), and the AUC of validation set were 0.782 (95% CI, 0.713–0.851; sensitivity = 0.73 and specificity = 0.71) and 0.907 (95% CI, 0.854–0.960; sensitivity = 0.88 and specificity = 0.82), respectively (Figure 2). The AUCs of SVM and LR had no significant difference in both datasets. The combined model showed a better performance than radiomics-based model in both training set and validation set (Figure 2). In addition, we summarized four indicators (precision, recall, f1-score, support) for each classifier in Tables 4, 5.

**Table 1:** Clinical characteristics and radiological signs in training and validation set.

Dataset	Training set			Validation set		
	AVM	Hypertension	*p*	AVM	Hypertension	*p*
Patients, No. (%)	207 (51.9%)	192 (48.1%)	–	89 (51.7%)	83 (48.3%)	–
Age, median (Range)	34 (7–83)	59 (24–93)	<0.001	33 (5–83)	59 (33–94)	<0.001
Sex, M/F	139/68	126/66	0.52	58/31	50/33	0.50
Calcification, No. (%)	47 (29.4%)	2 (1.1%)	<0.001	15 (20.3%)	2 (2.5%)	<0.001

No. (%), the numbers before parentheses represent the actual numbers and the numbers.

**Table 2:** ROC results with SVM and LR classifiers of combined model and radiomics-based model in training set.

Classifiers	Model	AUC	95% CI	Sensitivity	Specificity
SVM	Combined model	0.976	0.953–0.999	0.95	0.95
	Radiomics-based model	0.894	0.858–0.932	0.83	0.82
LR	Combined model	0.981	0.953–0.999	0.95	0.95
	Radiomics-based model	0.904	0.866–0.942	0.82	0.82

**Table 3:** ROC results with SVM and LR classifiers of combined model and radiomics-based model in validation set.

Classifiers	Model	AUC	95% CI	Sensitivity	Specificity
SVM	Combined model	0.896	0.841–0.951	0.88	0.8
	Radiomics-based model	0.774	0.705–0.843	0.72	0.71
LR	Combined model	0.907	0.854–0.960	0.88	0.82
	Radiomics-based model	0.782	0.713–0.851	0.73	0.71

**Table 4:** The results of precision, sensitivity, F1-score, support of combined model and radiomics-based model in training set.

Indicators	Combined model		Radiomics-based model	
	SVM	LR	SVM	LR
Precision	0.95	0.95	0.83	0.83
Sensitivity	0.95	0.94	0.83	0.82
F1-score	0.95	0.94	0.83	0.83
Support	207	207	207	207

**Table 5:** The results of precision, sensitivity, F1-score, support of combined model and radiomics-based model in validation set.

Indicators	Combined model		Radiomics-based model	
	SVM	LR	SVM	LR
Precision	0.82	0.84	0.73	0.73
Sensitivity	0.88	0.88	0.72	0.73
F1-score	0.85	0.86	0.72	0.73
Support	89	89	89	89

## Discussion

In this study, we extracted and analyzed 1,688 radiomics features to provide a more comprehensive depiction of the internal heterogeneity of hematomas. After processing, a total of 67 features were finally selected, including the radiomics features of the shape, first order, gray-level co-occurrence matrix (GLCM), gray-level size zone matrix (GLSZM), gray level run length matrix (GLRLM), gray level dependence matrix (GLDM), and neighbouring gray tone difference matrix (NGTDM). Then, we constructed and evaluated two predictive models (radiomics-based model and combined model) to non-invasively distinguish AVM related hematomas from hypertensive intracerebral hematomas using non-enhanced CT based radiomics. Compared to radiomics-based model, the combined model using radiomics, age and calcification showed a better performance and hold great potential for personalized clinical decision.

**Figure 3:**
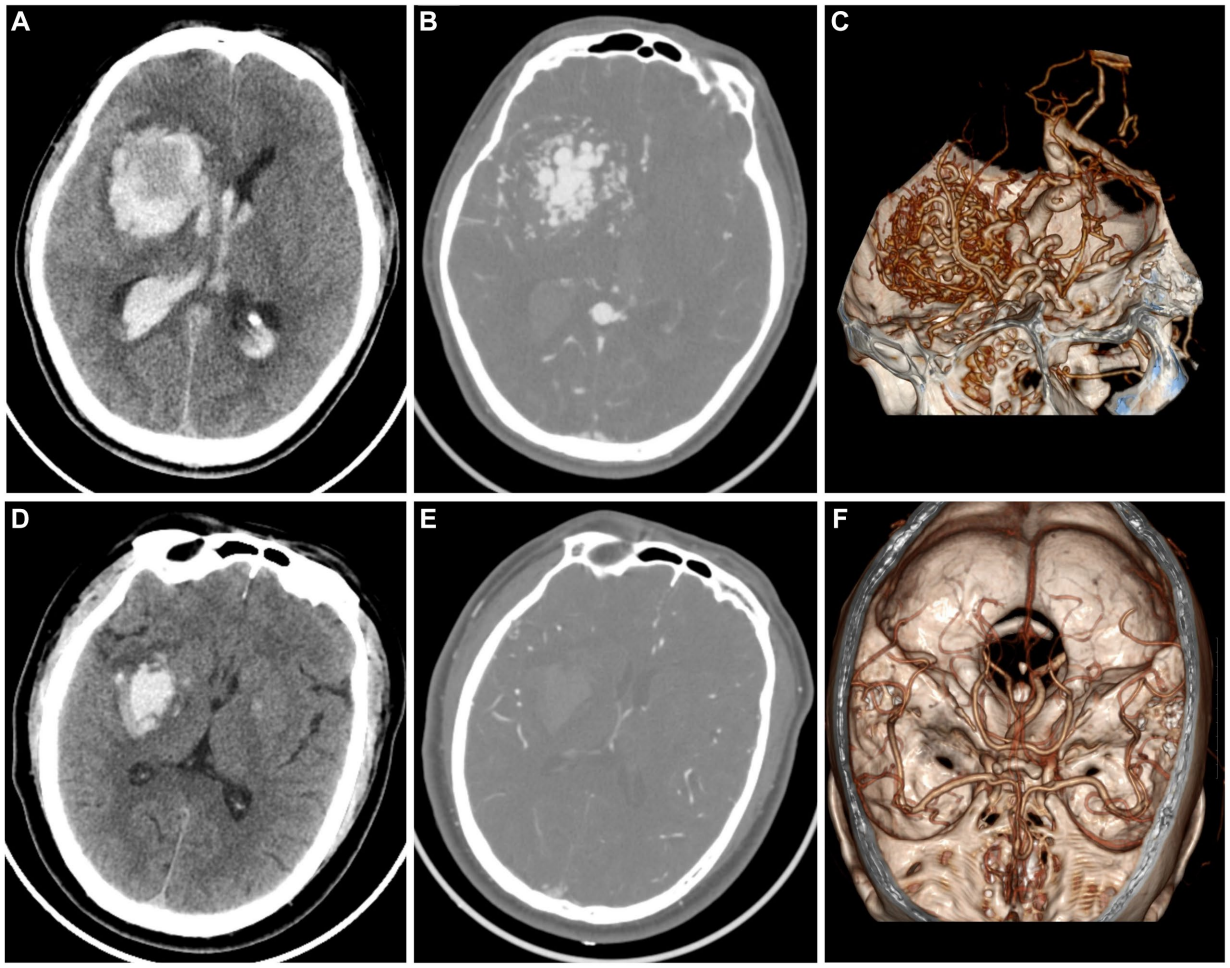
A case of AVM related hematoma, man, 42 years old, presented with unconsciousness for 4 h. Non-enhanced CT showed a hematoma in the right basal ganglia **(A)**. CT angiography **(B)** and volume rendering technique **(C)** showed a malformed vascular mass. A case of hypertensive intracerebral hematoma, man, 47 years old, presented with slurred speech with left limb weakness for 5 h. Non-enhanced CT showed a hematoma in the right basal ganglia **(D)**. CT angiography **(E)** and volume rendering technique **(F)** showed no abnormality.

The timely implementation of surgical or interventional procedures has a positive impact on the outcomes of patients with AVM, whereas delayed intervention beyond 48 h after symptom onset may result in worsened effects. Therefore, prompt screening for further operation is recommended for patients with AVM instead of conservative treatment as those with hypertensive intracerebral hematomas. However, it is challenging for the naked eye by radiologists to discriminate AVM related hematomas from hypertensive intracerebral hematomas on nonenhanced CT (Figure 3). Older age, and a history of hypertension are commonly used as diagnostic consideration for hypertensive intracerebral hematomas. However, many patients of intracerebral hemorrhage arrive at the emergency room in a coma and cannot even provide their age or history of hypertension. In addition, cerebral angiography studies suggest that these above clinical and imaging features may not always be reliable indicators (Josephson et al., 2014; van Asch et al., 2015). Therefore, radiomics-based classification model actually provided additional supplementary diagnostic for identifying AVM related hematomas.

Radiomics, utilizing diverse machine learning techniques to construct predictive models, can non-invasively reflect the internal heterogeneity of lesions for early diagnosis, differential diagnosis, and prognostic predictions. This approach has been extensively investigated in various cancer studies (Artzi et al., 2019; Wang et al., 2019; Li et al., 2022). AVM related hematomas are more likely to be irregular and heterogeneous due to the presence of calcification and malformed vasculatures embedded in the hematomas, whereas hypertensive intracerebral hematomas are more likely to have a uniform shape. These imaging feature can be reflected by radiomics features. So far, radiomics analysis of vascular diseases has primarily focused on identifying and stratifying the stability of vessel plaques (Shi et al., 2018; Kolossváry et al., 2019), predicting cerebral hematomas expansion (Xie et al., 2020; Xu W. et al., 2020), and identifying tumorous intracerebral hemorrhages (Choi et al., 2015; Nawabi et al., 2020). These previous studies have demonstrated that radiomics features may have the potential to objectively quantify the shape of hematomas and the heterogeneity of hematomas. In this study, three radiomics features belonging to the shape and many radiomics features belonging to the first order, GLCM, GLDM, GLRLM, GLSZM, and NGTDM that described the heterogeneity of the hematoma may be associated with AVM related hematomas (Supplementary Table S2). The non-enhanced CT based radiomics model may present potential benefits such as reduced contrast and radiation exposure, as well as faster treatment times that could lead to improved outcomes. In our study, we found that age and calcification had significant differences between AVM related hematomas and hypertensive intracerebral hematomas. Patients with AVM related hematomas were younger than patients with hypertensive hematomas. Calcification was more commonly seen in AVM related hematomas than hypertensive hematomas. Therefore, we further developed a combined model which combined with selected radiomics features, age and calcification. Expectedly, the combined model showed a satisfactory predictive performance for discrimination of AVM related hematomas from hypertensive intracerebral hematomas. In the future, the combined model based on non-enhanced CT may present an appealing complementary alternative for emergency department physician in accurately identifying AVM related hematomas.

There were several limitations in this study. Firstly, this study lacked independent external validation cohort. In the future, external multi-center validation cohorts are needed. Secondly, this study is a retrospective study. In the future, prospective cohorts should be further collected to validate the radiomics model. Thirdly, although hypertension and AVM are the two most common causes of spontaneous intraparenchymal hematomas, several relatively rare causes, such as cerebral amyloid angiopathy are not included in our study. Therefore, the constructed model in our study can only be applied specifically for distinguishing the two most common causes of spontaneous intraparenchymal hematomas. Lastly, in this study, the semiautomatic method was used for hematomas segmentation. Future research could investigate the potential of artificial intelligence in facilitating rapid and comprehensive segmentation (Xu J. et al., 2020; Yao et al., 2020).

In summary, non-enhanced CT is a non-invasive and time-saving technique that does not require contrast injection. Non-enhanced CT based radiomics and combined models were developed and validated in this study, which provides a valuable tool for the individualized risk prediction of AVM related hematomas, which may serve as a promising tool to complement the conventional procedures for the clinical decision-making process in the future.

## Data availability statement

The original contributions presented in the study are included in the article/Supplementary material, further inquiries can be directed to the corresponding author.

## Ethics statement

The studies involving humans were approved by the Ethics Committee of the Second Affiliated Hospital, Zhejiang University School of Medicine. The studies were conducted in accordance with the local legislation and institutional requirements. The participants provided their written informed consent to participate in this study.

## Author contributions

HX: Data curation, Formal analysis, Investigation, Resources, Writing – original draft. FD: Formal analysis, Investigation, Methodology, Writing – original draft. RZ: Investigation, Methodology, Writing – original draft. XY: Formal analysis, Investigation, Writing – original draft. PX: Data curation, Investigation, Writing – original draft. YT: Data curation, Investigation, Writing – original draft. PH: Supervision, Visualization, Writing – review & editing. CW: Conceptualization, Funding acquisition, Methodology, Supervision, Validation, Visualization, Writing – review & editing.
