# Supplementary material for: Building nonenhanced CT based radiomics model in discriminating arteriovenous malformation related hematomas from hypertensive intracerebral hematomas
Source: Front Neurosci. 2023 Nov 28;17:1284560. doi: 10.3389/fnins.2023.1284560 (PMC10713806; doi:10.3389/fnins.2023.1284560)
Supplement: Supplementary file 1 [file Table_1.DOCX]

**Supplementary Materials**

**Table S1. Details of the 1688 radiomic features**

| InterquartileRange | firstorder | original |
| --- | --- | --- |
| Skewness | firstorder | original |
| Uniformity | firstorder | original |
| Median | firstorder | original |
| Energy | firstorder | original |
| RobustMeanAbsoluteDeviation | firstorder | original |
| MeanAbsoluteDeviation | firstorder | original |
| TotalEnergy | firstorder | original |
| Maximum | firstorder | original |
| RootMeanSquared | firstorder | original |
| 90Percentile | firstorder | original |
| Minimum | firstorder | original |
| Entropy | firstorder | original |
| Range | firstorder | original |
| Variance | firstorder | original |
| 10Percentile | firstorder | original |
| Kurtosis | firstorder | original |
| Mean | firstorder | original |
| VoxelVolume | shape | original |
| Maximum3DDiameter | shape | original |
| MeshVolume | shape | original |
| MajorAxisLength | shape | original |
| Sphericity | shape | original |
| LeastAxisLength | shape | original |
| Elongation | shape | original |
| SurfaceVolumeRatio | shape | original |
| Maximum2DDiameterSlice | shape | original |
| Flatness | shape | original |
| SurfaceArea | shape | original |
| MinorAxisLength | shape | original |
| Maximum2DDiameterColumn | shape | original |
| Maximum2DDiameterRow | shape | original |
| JointAverage | glcm | original |
| SumAverage | glcm | original |
| JointEntropy | glcm | original |
| ClusterShade | glcm | original |
| MaximumProbability | glcm | original |
| Idmn | glcm | original |
| JointEnergy | glcm | original |
| Contrast | glcm | original |
| DifferenceEntropy | glcm | original |
| InverseVariance | glcm | original |
| DifferenceVariance | glcm | original |
| Idn | glcm | original |
| Idm | glcm | original |
| Correlation | glcm | original |
| Autocorrelation | glcm | original |
| SumEntropy | glcm | original |
| MCC | glcm | original |
| SumSquares | glcm | original |
| ClusterProminence | glcm | original |
| Imc2 | glcm | original |
| Imc1 | glcm | original |
| DifferenceAverage | glcm | original |
| Id | glcm | original |
| ClusterTendency | glcm | original |
| GrayLevelVariance | gldm | original |
| HighGrayLevelEmphasis | gldm | original |
| DependenceEntropy | gldm | original |
| DependenceNonUniformity | gldm | original |
| GrayLevelNonUniformity | gldm | original |
| SmallDependenceEmphasis | gldm | original |
| SmallDependenceHighGrayLevelEmphasis | gldm | original |
| DependenceNonUniformityNormalized | gldm | original |
| LargeDependenceEmphasis | gldm | original |
| LargeDependenceLowGrayLevelEmphasis | gldm | original |
| DependenceVariance | gldm | original |
| LargeDependenceHighGrayLevelEmphasis | gldm | original |
| SmallDependenceLowGrayLevelEmphasis | gldm | original |
| LowGrayLevelEmphasis | gldm | original |
| ShortRunLowGrayLevelEmphasis | glrlm | original |
| GrayLevelVariance | glrlm | original |
| LowGrayLevelRunEmphasis | glrlm | original |
| GrayLevelNonUniformityNormalized | glrlm | original |
| RunVariance | glrlm | original |
| GrayLevelNonUniformity | glrlm | original |
| LongRunEmphasis | glrlm | original |
| ShortRunHighGrayLevelEmphasis | glrlm | original |
| RunLengthNonUniformity | glrlm | original |
| ShortRunEmphasis | glrlm | original |
| LongRunHighGrayLevelEmphasis | glrlm | original |
| RunPercentage | glrlm | original |
| LongRunLowGrayLevelEmphasis | glrlm | original |
| RunEntropy | glrlm | original |
| HighGrayLevelRunEmphasis | glrlm | original |
| RunLengthNonUniformityNormalized | glrlm | original |
| GrayLevelVariance | glszm | original |
| ZoneVariance | glszm | original |
| GrayLevelNonUniformityNormalized | glszm | original |
| SizeZoneNonUniformityNormalized | glszm | original |
| SizeZoneNonUniformity | glszm | original |
| GrayLevelNonUniformity | glszm | original |
| LargeAreaEmphasis | glszm | original |
| SmallAreaHighGrayLevelEmphasis | glszm | original |
| ZonePercentage | glszm | original |
| LargeAreaLowGrayLevelEmphasis | glszm | original |
| LargeAreaHighGrayLevelEmphasis | glszm | original |
| HighGrayLevelZoneEmphasis | glszm | original |
| SmallAreaEmphasis | glszm | original |
| LowGrayLevelZoneEmphasis | glszm | original |
| ZoneEntropy | glszm | original |
| SmallAreaLowGrayLevelEmphasis | glszm | original |
| Coarseness | ngtdm | original |
| Complexity | ngtdm | original |
| Strength | ngtdm | original |
| Contrast | ngtdm | original |
| Busyness | ngtdm | original |
| InterquartileRange | firstorder | logarithm |
| Skewness | firstorder | logarithm |
| Uniformity | firstorder | logarithm |
| Median | firstorder | logarithm |
| Energy | firstorder | logarithm |
| RobustMeanAbsoluteDeviation | firstorder | logarithm |
| MeanAbsoluteDeviation | firstorder | logarithm |
| TotalEnergy | firstorder | logarithm |
| Maximum | firstorder | logarithm |
| RootMeanSquared | firstorder | logarithm |
| 90Percentile | firstorder | logarithm |
| Minimum | firstorder | logarithm |
| Entropy | firstorder | logarithm |
| Range | firstorder | logarithm |
| Variance | firstorder | logarithm |
| 10Percentile | firstorder | logarithm |
| Kurtosis | firstorder | logarithm |
| Mean | firstorder | logarithm |
| JointAverage | glcm | logarithm |
| SumAverage | glcm | logarithm |
| JointEntropy | glcm | logarithm |
| ClusterShade | glcm | logarithm |
| MaximumProbability | glcm | logarithm |
| Idmn | glcm | logarithm |
| JointEnergy | glcm | logarithm |
| Contrast | glcm | logarithm |
| DifferenceEntropy | glcm | logarithm |
| InverseVariance | glcm | logarithm |
| DifferenceVariance | glcm | logarithm |
| Idn | glcm | logarithm |
| Idm | glcm | logarithm |
| Correlation | glcm | logarithm |
| Autocorrelation | glcm | logarithm |
| SumEntropy | glcm | logarithm |
| MCC | glcm | logarithm |
| SumSquares | glcm | logarithm |
| ClusterProminence | glcm | logarithm |
| Imc2 | glcm | logarithm |
| Imc1 | glcm | logarithm |
| DifferenceAverage | glcm | logarithm |
| Id | glcm | logarithm |
| ClusterTendency | glcm | logarithm |
| GrayLevelVariance | gldm | logarithm |
| HighGrayLevelEmphasis | gldm | logarithm |
| DependenceEntropy | gldm | logarithm |
| DependenceNonUniformity | gldm | logarithm |
| GrayLevelNonUniformity | gldm | logarithm |
| SmallDependenceEmphasis | gldm | logarithm |
| SmallDependenceHighGrayLevelEmphasis | gldm | logarithm |
| DependenceNonUniformityNormalized | gldm | logarithm |
| LargeDependenceEmphasis | gldm | logarithm |
| LargeDependenceLowGrayLevelEmphasis | gldm | logarithm |
| DependenceVariance | gldm | logarithm |
| LargeDependenceHighGrayLevelEmphasis | gldm | logarithm |
| SmallDependenceLowGrayLevelEmphasis | gldm | logarithm |
| LowGrayLevelEmphasis | gldm | logarithm |
| ShortRunLowGrayLevelEmphasis | glrlm | logarithm |
| GrayLevelVariance | glrlm | logarithm |
| LowGrayLevelRunEmphasis | glrlm | logarithm |
| GrayLevelNonUniformityNormalized | glrlm | logarithm |
| RunVariance | glrlm | logarithm |
| GrayLevelNonUniformity | glrlm | logarithm |
| LongRunEmphasis | glrlm | logarithm |
| ShortRunHighGrayLevelEmphasis | glrlm | logarithm |
| RunLengthNonUniformity | glrlm | logarithm |
| ShortRunEmphasis | glrlm | logarithm |
| LongRunHighGrayLevelEmphasis | glrlm | logarithm |
| RunPercentage | glrlm | logarithm |
| LongRunLowGrayLevelEmphasis | glrlm | logarithm |
| RunEntropy | glrlm | logarithm |
| HighGrayLevelRunEmphasis | glrlm | logarithm |
| RunLengthNonUniformityNormalized | glrlm | logarithm |
| GrayLevelVariance | glszm | logarithm |
| ZoneVariance | glszm | logarithm |
| GrayLevelNonUniformityNormalized | glszm | logarithm |
| SizeZoneNonUniformityNormalized | glszm | logarithm |
| SizeZoneNonUniformity | glszm | logarithm |
| GrayLevelNonUniformity | glszm | logarithm |
| LargeAreaEmphasis | glszm | logarithm |
| SmallAreaHighGrayLevelEmphasis | glszm | logarithm |
| ZonePercentage | glszm | logarithm |
| LargeAreaLowGrayLevelEmphasis | glszm | logarithm |
| LargeAreaHighGrayLevelEmphasis | glszm | logarithm |
| HighGrayLevelZoneEmphasis | glszm | logarithm |
| SmallAreaEmphasis | glszm | logarithm |
| LowGrayLevelZoneEmphasis | glszm | logarithm |
| ZoneEntropy | glszm | logarithm |
| SmallAreaLowGrayLevelEmphasis | glszm | logarithm |
| Coarseness | ngtdm | logarithm |
| Complexity | ngtdm | logarithm |
| Strength | ngtdm | logarithm |
| Contrast | ngtdm | logarithm |
| Busyness | ngtdm | logarithm |
| InterquartileRange | firstorder | exponential |
| Skewness | firstorder | exponential |
| Uniformity | firstorder | exponential |
| Median | firstorder | exponential |
| Energy | firstorder | exponential |
| RobustMeanAbsoluteDeviation | firstorder | exponential |
| MeanAbsoluteDeviation | firstorder | exponential |
| TotalEnergy | firstorder | exponential |
| Maximum | firstorder | exponential |
| RootMeanSquared | firstorder | exponential |
| 90Percentile | firstorder | exponential |
| Minimum | firstorder | exponential |
| Entropy | firstorder | exponential |
| Range | firstorder | exponential |
| Variance | firstorder | exponential |
| 10Percentile | firstorder | exponential |
| Kurtosis | firstorder | exponential |
| Mean | firstorder | exponential |
| JointAverage | glcm | exponential |
| SumAverage | glcm | exponential |
| JointEntropy | glcm | exponential |
| ClusterShade | glcm | exponential |
| MaximumProbability | glcm | exponential |
| Idmn | glcm | exponential |
| JointEnergy | glcm | exponential |
| Contrast | glcm | exponential |
| DifferenceEntropy | glcm | exponential |
| InverseVariance | glcm | exponential |
| DifferenceVariance | glcm | exponential |
| Idn | glcm | exponential |
| Idm | glcm | exponential |
| Correlation | glcm | exponential |
| Autocorrelation | glcm | exponential |
| SumEntropy | glcm | exponential |
| MCC | glcm | exponential |
| SumSquares | glcm | exponential |
| ClusterProminence | glcm | exponential |
| Imc2 | glcm | exponential |
| Imc1 | glcm | exponential |
| DifferenceAverage | glcm | exponential |
| Id | glcm | exponential |
| ClusterTendency | glcm | exponential |
| GrayLevelVariance | gldm | exponential |
| HighGrayLevelEmphasis | gldm | exponential |
| DependenceEntropy | gldm | exponential |
| DependenceNonUniformity | gldm | exponential |
| GrayLevelNonUniformity | gldm | exponential |
| SmallDependenceEmphasis | gldm | exponential |
| SmallDependenceHighGrayLevelEmphasis | gldm | exponential |
| DependenceNonUniformityNormalized | gldm | exponential |
| LargeDependenceEmphasis | gldm | exponential |
| LargeDependenceLowGrayLevelEmphasis | gldm | exponential |
| DependenceVariance | gldm | exponential |
| LargeDependenceHighGrayLevelEmphasis | gldm | exponential |
| SmallDependenceLowGrayLevelEmphasis | gldm | exponential |
| LowGrayLevelEmphasis | gldm | exponential |
| ShortRunLowGrayLevelEmphasis | glrlm | exponential |
| GrayLevelVariance | glrlm | exponential |
| LowGrayLevelRunEmphasis | glrlm | exponential |
| GrayLevelNonUniformityNormalized | glrlm | exponential |
| RunVariance | glrlm | exponential |
| GrayLevelNonUniformity | glrlm | exponential |
| LongRunEmphasis | glrlm | exponential |
| ShortRunHighGrayLevelEmphasis | glrlm | exponential |
| RunLengthNonUniformity | glrlm | exponential |
| ShortRunEmphasis | glrlm | exponential |
| LongRunHighGrayLevelEmphasis | glrlm | exponential |
| RunPercentage | glrlm | exponential |
| LongRunLowGrayLevelEmphasis | glrlm | exponential |
| RunEntropy | glrlm | exponential |
| HighGrayLevelRunEmphasis | glrlm | exponential |
| RunLengthNonUniformityNormalized | glrlm | exponential |
| GrayLevelVariance | glszm | exponential |
| ZoneVariance | glszm | exponential |
| GrayLevelNonUniformityNormalized | glszm | exponential |
| SizeZoneNonUniformityNormalized | glszm | exponential |
| SizeZoneNonUniformity | glszm | exponential |
| GrayLevelNonUniformity | glszm | exponential |
| LargeAreaEmphasis | glszm | exponential |
| SmallAreaHighGrayLevelEmphasis | glszm | exponential |
| ZonePercentage | glszm | exponential |
| LargeAreaLowGrayLevelEmphasis | glszm | exponential |
| LargeAreaHighGrayLevelEmphasis | glszm | exponential |
| HighGrayLevelZoneEmphasis | glszm | exponential |
| SmallAreaEmphasis | glszm | exponential |
| LowGrayLevelZoneEmphasis | glszm | exponential |
| ZoneEntropy | glszm | exponential |
| SmallAreaLowGrayLevelEmphasis | glszm | exponential |
| Coarseness | ngtdm | exponential |
| Complexity | ngtdm | exponential |
| Strength | ngtdm | exponential |
| Contrast | ngtdm | exponential |
| Busyness | ngtdm | exponential |
| InterquartileRange | firstorder | gradient |
| Skewness | firstorder | gradient |
| Uniformity | firstorder | gradient |
| Median | firstorder | gradient |
| Energy | firstorder | gradient |
| RobustMeanAbsoluteDeviation | firstorder | gradient |
| MeanAbsoluteDeviation | firstorder | gradient |
| TotalEnergy | firstorder | gradient |
| Maximum | firstorder | gradient |
| RootMeanSquared | firstorder | gradient |
| 90Percentile | firstorder | gradient |
| Minimum | firstorder | gradient |
| Entropy | firstorder | gradient |
| Range | firstorder | gradient |
| Variance | firstorder | gradient |
| 10Percentile | firstorder | gradient |
| Kurtosis | firstorder | gradient |
| Mean | firstorder | gradient |
| JointAverage | glcm | gradient |
| SumAverage | glcm | gradient |
| JointEntropy | glcm | gradient |
| ClusterShade | glcm | gradient |
| MaximumProbability | glcm | gradient |
| Idmn | glcm | gradient |
| JointEnergy | glcm | gradient |
| Contrast | glcm | gradient |
| DifferenceEntropy | glcm | gradient |
| InverseVariance | glcm | gradient |
| DifferenceVariance | glcm | gradient |
| Idn | glcm | gradient |
| Idm | glcm | gradient |
| Correlation | glcm | gradient |
| Autocorrelation | glcm | gradient |
| SumEntropy | glcm | gradient |
| MCC | glcm | gradient |
| SumSquares | glcm | gradient |
| ClusterProminence | glcm | gradient |
| Imc2 | glcm | gradient |
| Imc1 | glcm | gradient |
| DifferenceAverage | glcm | gradient |
| Id | glcm | gradient |
| ClusterTendency | glcm | gradient |
| GrayLevelVariance | gldm | gradient |
| HighGrayLevelEmphasis | gldm | gradient |
| DependenceEntropy | gldm | gradient |
| DependenceNonUniformity | gldm | gradient |
| GrayLevelNonUniformity | gldm | gradient |
| SmallDependenceEmphasis | gldm | gradient |
| SmallDependenceHighGrayLevelEmphasis | gldm | gradient |
| DependenceNonUniformityNormalized | gldm | gradient |
| LargeDependenceEmphasis | gldm | gradient |
| LargeDependenceLowGrayLevelEmphasis | gldm | gradient |
| DependenceVariance | gldm | gradient |
| LargeDependenceHighGrayLevelEmphasis | gldm | gradient |
| SmallDependenceLowGrayLevelEmphasis | gldm | gradient |
| LowGrayLevelEmphasis | gldm | gradient |
| ShortRunLowGrayLevelEmphasis | glrlm | gradient |
| GrayLevelVariance | glrlm | gradient |
| LowGrayLevelRunEmphasis | glrlm | gradient |
| GrayLevelNonUniformityNormalized | glrlm | gradient |
| RunVariance | glrlm | gradient |
| GrayLevelNonUniformity | glrlm | gradient |
| LongRunEmphasis | glrlm | gradient |
| ShortRunHighGrayLevelEmphasis | glrlm | gradient |
| RunLengthNonUniformity | glrlm | gradient |
| ShortRunEmphasis | glrlm | gradient |
| LongRunHighGrayLevelEmphasis | glrlm | gradient |
| RunPercentage | glrlm | gradient |
| LongRunLowGrayLevelEmphasis | glrlm | gradient |
| RunEntropy | glrlm | gradient |
| HighGrayLevelRunEmphasis | glrlm | gradient |
| RunLengthNonUniformityNormalized | glrlm | gradient |
| GrayLevelVariance | glszm | gradient |
| ZoneVariance | glszm | gradient |
| GrayLevelNonUniformityNormalized | glszm | gradient |
| SizeZoneNonUniformityNormalized | glszm | gradient |
| SizeZoneNonUniformity | glszm | gradient |
| GrayLevelNonUniformity | glszm | gradient |
| LargeAreaEmphasis | glszm | gradient |
| SmallAreaHighGrayLevelEmphasis | glszm | gradient |
| ZonePercentage | glszm | gradient |
| LargeAreaLowGrayLevelEmphasis | glszm | gradient |
| LargeAreaHighGrayLevelEmphasis | glszm | gradient |
| HighGrayLevelZoneEmphasis | glszm | gradient |
| SmallAreaEmphasis | glszm | gradient |
| LowGrayLevelZoneEmphasis | glszm | gradient |
| ZoneEntropy | glszm | gradient |
| SmallAreaLowGrayLevelEmphasis | glszm | gradient |
| Coarseness | ngtdm | gradient |
| Complexity | ngtdm | gradient |
| Strength | ngtdm | gradient |
| Contrast | ngtdm | gradient |
| Busyness | ngtdm | gradient |
| InterquartileRange | firstorder | square |
| Skewness | firstorder | square |
| Uniformity | firstorder | square |
| Median | firstorder | square |
| Energy | firstorder | square |
| RobustMeanAbsoluteDeviation | firstorder | square |
| MeanAbsoluteDeviation | firstorder | square |
| TotalEnergy | firstorder | square |
| Maximum | firstorder | square |
| RootMeanSquared | firstorder | square |
| 90Percentile | firstorder | square |
| Minimum | firstorder | square |
| Entropy | firstorder | square |
| Range | firstorder | square |
| Variance | firstorder | square |
| 10Percentile | firstorder | square |
| Kurtosis | firstorder | square |
| Mean | firstorder | square |
| JointAverage | glcm | square |
| SumAverage | glcm | square |
| JointEntropy | glcm | square |
| ClusterShade | glcm | square |
| MaximumProbability | glcm | square |
| Idmn | glcm | square |
| JointEnergy | glcm | square |
| Contrast | glcm | square |
| DifferenceEntropy | glcm | square |
| InverseVariance | glcm | square |
| DifferenceVariance | glcm | square |
| Idn | glcm | square |
| Idm | glcm | square |
| Correlation | glcm | square |
| Autocorrelation | glcm | square |
| SumEntropy | glcm | square |
| MCC | glcm | square |
| SumSquares | glcm | square |
| ClusterProminence | glcm | square |
| Imc2 | glcm | square |
| Imc1 | glcm | square |
| DifferenceAverage | glcm | square |
| Id | glcm | square |
| ClusterTendency | glcm | square |
| GrayLevelVariance | gldm | square |
| HighGrayLevelEmphasis | gldm | square |
| DependenceEntropy | gldm | square |
| DependenceNonUniformity | gldm | square |
| GrayLevelNonUniformity | gldm | square |
| SmallDependenceEmphasis | gldm | square |
| SmallDependenceHighGrayLevelEmphasis | gldm | square |
| DependenceNonUniformityNormalized | gldm | square |
| LargeDependenceEmphasis | gldm | square |
| LargeDependenceLowGrayLevelEmphasis | gldm | square |
| DependenceVariance | gldm | square |
| LargeDependenceHighGrayLevelEmphasis | gldm | square |
| SmallDependenceLowGrayLevelEmphasis | gldm | square |
| LowGrayLevelEmphasis | gldm | square |
| ShortRunLowGrayLevelEmphasis | glrlm | square |
| GrayLevelVariance | glrlm | square |
| LowGrayLevelRunEmphasis | glrlm | square |
| GrayLevelNonUniformityNormalized | glrlm | square |
| RunVariance | glrlm | square |
| GrayLevelNonUniformity | glrlm | square |
| LongRunEmphasis | glrlm | square |
| ShortRunHighGrayLevelEmphasis | glrlm | square |
| RunLengthNonUniformity | glrlm | square |
| ShortRunEmphasis | glrlm | square |
| LongRunHighGrayLevelEmphasis | glrlm | square |
| RunPercentage | glrlm | square |
| LongRunLowGrayLevelEmphasis | glrlm | square |
| RunEntropy | glrlm | square |
| HighGrayLevelRunEmphasis | glrlm | square |
| RunLengthNonUniformityNormalized | glrlm | square |
| GrayLevelVariance | glszm | square |
| ZoneVariance | glszm | square |
| GrayLevelNonUniformityNormalized | glszm | square |
| SizeZoneNonUniformityNormalized | glszm | square |
| SizeZoneNonUniformity | glszm | square |
| GrayLevelNonUniformity | glszm | square |
| LargeAreaEmphasis | glszm | square |
| SmallAreaHighGrayLevelEmphasis | glszm | square |
| ZonePercentage | glszm | square |
| LargeAreaLowGrayLevelEmphasis | glszm | square |
| LargeAreaHighGrayLevelEmphasis | glszm | square |
| HighGrayLevelZoneEmphasis | glszm | square |
| SmallAreaEmphasis | glszm | square |
| LowGrayLevelZoneEmphasis | glszm | square |
| ZoneEntropy | glszm | square |
| SmallAreaLowGrayLevelEmphasis | glszm | square |
| Coarseness | ngtdm | square |
| Complexity | ngtdm | square |
| Strength | ngtdm | square |
| Contrast | ngtdm | square |
| Busyness | ngtdm | square |
| InterquartileRange | firstorder | squareroot |
| Skewness | firstorder | squareroot |
| Uniformity | firstorder | squareroot |
| Median | firstorder | squareroot |
| Energy | firstorder | squareroot |
| RobustMeanAbsoluteDeviation | firstorder | squareroot |
| MeanAbsoluteDeviation | firstorder | squareroot |
| TotalEnergy | firstorder | squareroot |
| Maximum | firstorder | squareroot |
| RootMeanSquared | firstorder | squareroot |
| 90Percentile | firstorder | squareroot |
| Minimum | firstorder | squareroot |
| Entropy | firstorder | squareroot |
| Range | firstorder | squareroot |
| Variance | firstorder | squareroot |
| 10Percentile | firstorder | squareroot |
| Kurtosis | firstorder | squareroot |
| Mean | firstorder | squareroot |
| JointAverage | glcm | squareroot |
| SumAverage | glcm | squareroot |
| JointEntropy | glcm | squareroot |
| ClusterShade | glcm | squareroot |
| MaximumProbability | glcm | squareroot |
| Idmn | glcm | squareroot |
| JointEnergy | glcm | squareroot |
| Contrast | glcm | squareroot |
| DifferenceEntropy | glcm | squareroot |
| InverseVariance | glcm | squareroot |
| DifferenceVariance | glcm | squareroot |
| Idn | glcm | squareroot |
| Idm | glcm | squareroot |
| Correlation | glcm | squareroot |
| Autocorrelation | glcm | squareroot |
| SumEntropy | glcm | squareroot |
| MCC | glcm | squareroot |
| SumSquares | glcm | squareroot |
| ClusterProminence | glcm | squareroot |
| Imc2 | glcm | squareroot |
| Imc1 | glcm | squareroot |
| DifferenceAverage | glcm | squareroot |
| Id | glcm | squareroot |
| ClusterTendency | glcm | squareroot |
| GrayLevelVariance | gldm | squareroot |
| HighGrayLevelEmphasis | gldm | squareroot |
| DependenceEntropy | gldm | squareroot |
| DependenceNonUniformity | gldm | squareroot |
| GrayLevelNonUniformity | gldm | squareroot |
| SmallDependenceEmphasis | gldm | squareroot |
| SmallDependenceHighGrayLevelEmphasis | gldm | squareroot |
| DependenceNonUniformityNormalized | gldm | squareroot |
| LargeDependenceEmphasis | gldm | squareroot |
| LargeDependenceLowGrayLevelEmphasis | gldm | squareroot |
| DependenceVariance | gldm | squareroot |
| LargeDependenceHighGrayLevelEmphasis | gldm | squareroot |
| SmallDependenceLowGrayLevelEmphasis | gldm | squareroot |
| LowGrayLevelEmphasis | gldm | squareroot |
| ShortRunLowGrayLevelEmphasis | glrlm | squareroot |
| GrayLevelVariance | glrlm | squareroot |
| LowGrayLevelRunEmphasis | glrlm | squareroot |
| GrayLevelNonUniformityNormalized | glrlm | squareroot |
| RunVariance | glrlm | squareroot |
| GrayLevelNonUniformity | glrlm | squareroot |
| LongRunEmphasis | glrlm | squareroot |
| ShortRunHighGrayLevelEmphasis | glrlm | squareroot |
| RunLengthNonUniformity | glrlm | squareroot |
| ShortRunEmphasis | glrlm | squareroot |
| LongRunHighGrayLevelEmphasis | glrlm | squareroot |
| RunPercentage | glrlm | squareroot |
| LongRunLowGrayLevelEmphasis | glrlm | squareroot |
| RunEntropy | glrlm | squareroot |
| HighGrayLevelRunEmphasis | glrlm | squareroot |
| RunLengthNonUniformityNormalized | glrlm | squareroot |
| GrayLevelVariance | glszm | squareroot |
| ZoneVariance | glszm | squareroot |
| GrayLevelNonUniformityNormalized | glszm | squareroot |
| SizeZoneNonUniformityNormalized | glszm | squareroot |
| SizeZoneNonUniformity | glszm | squareroot |
| GrayLevelNonUniformity | glszm | squareroot |
| LargeAreaEmphasis | glszm | squareroot |
| SmallAreaHighGrayLevelEmphasis | glszm | squareroot |
| ZonePercentage | glszm | squareroot |
| LargeAreaLowGrayLevelEmphasis | glszm | squareroot |
| LargeAreaHighGrayLevelEmphasis | glszm | squareroot |
| HighGrayLevelZoneEmphasis | glszm | squareroot |
| SmallAreaEmphasis | glszm | squareroot |
| LowGrayLevelZoneEmphasis | glszm | squareroot |
| ZoneEntropy | glszm | squareroot |
| SmallAreaLowGrayLevelEmphasis | glszm | squareroot |
| Coarseness | ngtdm | squareroot |
| Complexity | ngtdm | squareroot |
| Strength | ngtdm | squareroot |
| Contrast | ngtdm | squareroot |
| Busyness | ngtdm | squareroot |
| InterquartileRange | firstorder | lbp-2D |
| Skewness | firstorder | lbp-2D |
| Uniformity | firstorder | lbp-2D |
| Median | firstorder | lbp-2D |
| Energy | firstorder | lbp-2D |
| RobustMeanAbsoluteDeviation | firstorder | lbp-2D |
| MeanAbsoluteDeviation | firstorder | lbp-2D |
| TotalEnergy | firstorder | lbp-2D |
| Maximum | firstorder | lbp-2D |
| RootMeanSquared | firstorder | lbp-2D |
| 90Percentile | firstorder | lbp-2D |
| Minimum | firstorder | lbp-2D |
| Entropy | firstorder | lbp-2D |
| Range | firstorder | lbp-2D |
| Variance | firstorder | lbp-2D |
| 10Percentile | firstorder | lbp-2D |
| Kurtosis | firstorder | lbp-2D |
| Mean | firstorder | lbp-2D |
| JointAverage | glcm | lbp-2D |
| SumAverage | glcm | lbp-2D |
| JointEntropy | glcm | lbp-2D |
| ClusterShade | glcm | lbp-2D |
| MaximumProbability | glcm | lbp-2D |
| Idmn | glcm | lbp-2D |
| JointEnergy | glcm | lbp-2D |
| Contrast | glcm | lbp-2D |
| DifferenceEntropy | glcm | lbp-2D |
| InverseVariance | glcm | lbp-2D |
| DifferenceVariance | glcm | lbp-2D |
| Idn | glcm | lbp-2D |
| Idm | glcm | lbp-2D |
| Correlation | glcm | lbp-2D |
| Autocorrelation | glcm | lbp-2D |
| SumEntropy | glcm | lbp-2D |
| MCC | glcm | lbp-2D |
| SumSquares | glcm | lbp-2D |
| ClusterProminence | glcm | lbp-2D |
| Imc2 | glcm | lbp-2D |
| Imc1 | glcm | lbp-2D |
| DifferenceAverage | glcm | lbp-2D |
| Id | glcm | lbp-2D |
| ClusterTendency | glcm | lbp-2D |
| GrayLevelVariance | gldm | lbp-2D |
| HighGrayLevelEmphasis | gldm | lbp-2D |
| DependenceEntropy | gldm | lbp-2D |
| DependenceNonUniformity | gldm | lbp-2D |
| GrayLevelNonUniformity | gldm | lbp-2D |
| SmallDependenceEmphasis | gldm | lbp-2D |
| SmallDependenceHighGrayLevelEmphasis | gldm | lbp-2D |
| DependenceNonUniformityNormalized | gldm | lbp-2D |
| LargeDependenceEmphasis | gldm | lbp-2D |
| LargeDependenceLowGrayLevelEmphasis | gldm | lbp-2D |
| DependenceVariance | gldm | lbp-2D |
| LargeDependenceHighGrayLevelEmphasis | gldm | lbp-2D |
| SmallDependenceLowGrayLevelEmphasis | gldm | lbp-2D |
| LowGrayLevelEmphasis | gldm | lbp-2D |
| ShortRunLowGrayLevelEmphasis | glrlm | lbp-2D |
| GrayLevelVariance | glrlm | lbp-2D |
| LowGrayLevelRunEmphasis | glrlm | lbp-2D |
| GrayLevelNonUniformityNormalized | glrlm | lbp-2D |
| RunVariance | glrlm | lbp-2D |
| GrayLevelNonUniformity | glrlm | lbp-2D |
| LongRunEmphasis | glrlm | lbp-2D |
| ShortRunHighGrayLevelEmphasis | glrlm | lbp-2D |
| RunLengthNonUniformity | glrlm | lbp-2D |
| ShortRunEmphasis | glrlm | lbp-2D |
| LongRunHighGrayLevelEmphasis | glrlm | lbp-2D |
| RunPercentage | glrlm | lbp-2D |
| LongRunLowGrayLevelEmphasis | glrlm | lbp-2D |
| RunEntropy | glrlm | lbp-2D |
| HighGrayLevelRunEmphasis | glrlm | lbp-2D |
| RunLengthNonUniformityNormalized | glrlm | lbp-2D |
| GrayLevelVariance | glszm | lbp-2D |
| ZoneVariance | glszm | lbp-2D |
| GrayLevelNonUniformityNormalized | glszm | lbp-2D |
| SizeZoneNonUniformityNormalized | glszm | lbp-2D |
| SizeZoneNonUniformity | glszm | lbp-2D |
| GrayLevelNonUniformity | glszm | lbp-2D |
| LargeAreaEmphasis | glszm | lbp-2D |
| SmallAreaHighGrayLevelEmphasis | glszm | lbp-2D |
| ZonePercentage | glszm | lbp-2D |
| LargeAreaLowGrayLevelEmphasis | glszm | lbp-2D |
| LargeAreaHighGrayLevelEmphasis | glszm | lbp-2D |
| HighGrayLevelZoneEmphasis | glszm | lbp-2D |
| SmallAreaEmphasis | glszm | lbp-2D |
| LowGrayLevelZoneEmphasis | glszm | lbp-2D |
| ZoneEntropy | glszm | lbp-2D |
| SmallAreaLowGrayLevelEmphasis | glszm | lbp-2D |
| Coarseness | ngtdm | lbp-2D |
| Complexity | ngtdm | lbp-2D |
| Strength | ngtdm | lbp-2D |
| Contrast | ngtdm | lbp-2D |
| Busyness | ngtdm | lbp-2D |
| InterquartileRange | firstorder | lbp-3D-m1 |
| Skewness | firstorder | lbp-3D-m1 |
| Uniformity | firstorder | lbp-3D-m1 |
| Median | firstorder | lbp-3D-m1 |
| Energy | firstorder | lbp-3D-m1 |
| RobustMeanAbsoluteDeviation | firstorder | lbp-3D-m1 |
| MeanAbsoluteDeviation | firstorder | lbp-3D-m1 |
| TotalEnergy | firstorder | lbp-3D-m1 |
| Maximum | firstorder | lbp-3D-m1 |
| RootMeanSquared | firstorder | lbp-3D-m1 |
| 90Percentile | firstorder | lbp-3D-m1 |
| Minimum | firstorder | lbp-3D-m1 |
| Entropy | firstorder | lbp-3D-m1 |
| Range | firstorder | lbp-3D-m1 |
| Variance | firstorder | lbp-3D-m1 |
| 10Percentile | firstorder | lbp-3D-m1 |
| Kurtosis | firstorder | lbp-3D-m1 |
| Mean | firstorder | lbp-3D-m1 |
| JointAverage | glcm | lbp-3D-m1 |
| SumAverage | glcm | lbp-3D-m1 |
| JointEntropy | glcm | lbp-3D-m1 |
| ClusterShade | glcm | lbp-3D-m1 |
| MaximumProbability | glcm | lbp-3D-m1 |
| Idmn | glcm | lbp-3D-m1 |
| JointEnergy | glcm | lbp-3D-m1 |
| Contrast | glcm | lbp-3D-m1 |
| DifferenceEntropy | glcm | lbp-3D-m1 |
| InverseVariance | glcm | lbp-3D-m1 |
| DifferenceVariance | glcm | lbp-3D-m1 |
| Idn | glcm | lbp-3D-m1 |
| Idm | glcm | lbp-3D-m1 |
| Correlation | glcm | lbp-3D-m1 |
| Autocorrelation | glcm | lbp-3D-m1 |
| SumEntropy | glcm | lbp-3D-m1 |
| MCC | glcm | lbp-3D-m1 |
| SumSquares | glcm | lbp-3D-m1 |
| ClusterProminence | glcm | lbp-3D-m1 |
| Imc2 | glcm | lbp-3D-m1 |
| Imc1 | glcm | lbp-3D-m1 |
| DifferenceAverage | glcm | lbp-3D-m1 |
| Id | glcm | lbp-3D-m1 |
| ClusterTendency | glcm | lbp-3D-m1 |
| GrayLevelVariance | gldm | lbp-3D-m1 |
| HighGrayLevelEmphasis | gldm | lbp-3D-m1 |
| DependenceEntropy | gldm | lbp-3D-m1 |
| DependenceNonUniformity | gldm | lbp-3D-m1 |
| GrayLevelNonUniformity | gldm | lbp-3D-m1 |
| SmallDependenceEmphasis | gldm | lbp-3D-m1 |
| SmallDependenceHighGrayLevelEmphasis | gldm | lbp-3D-m1 |
| DependenceNonUniformityNormalized | gldm | lbp-3D-m1 |
| LargeDependenceEmphasis | gldm | lbp-3D-m1 |
| LargeDependenceLowGrayLevelEmphasis | gldm | lbp-3D-m1 |
| DependenceVariance | gldm | lbp-3D-m1 |
| LargeDependenceHighGrayLevelEmphasis | gldm | lbp-3D-m1 |
| SmallDependenceLowGrayLevelEmphasis | gldm | lbp-3D-m1 |
| LowGrayLevelEmphasis | gldm | lbp-3D-m1 |
| ShortRunLowGrayLevelEmphasis | glrlm | lbp-3D-m1 |
| GrayLevelVariance | glrlm | lbp-3D-m1 |
| LowGrayLevelRunEmphasis | glrlm | lbp-3D-m1 |
| GrayLevelNonUniformityNormalized | glrlm | lbp-3D-m1 |
| RunVariance | glrlm | lbp-3D-m1 |
| GrayLevelNonUniformity | glrlm | lbp-3D-m1 |
| LongRunEmphasis | glrlm | lbp-3D-m1 |
| ShortRunHighGrayLevelEmphasis | glrlm | lbp-3D-m1 |
| RunLengthNonUniformity | glrlm | lbp-3D-m1 |
| ShortRunEmphasis | glrlm | lbp-3D-m1 |
| LongRunHighGrayLevelEmphasis | glrlm | lbp-3D-m1 |
| RunPercentage | glrlm | lbp-3D-m1 |
| LongRunLowGrayLevelEmphasis | glrlm | lbp-3D-m1 |
| RunEntropy | glrlm | lbp-3D-m1 |
| HighGrayLevelRunEmphasis | glrlm | lbp-3D-m1 |
| RunLengthNonUniformityNormalized | glrlm | lbp-3D-m1 |
| GrayLevelVariance | glszm | lbp-3D-m1 |
| ZoneVariance | glszm | lbp-3D-m1 |
| GrayLevelNonUniformityNormalized | glszm | lbp-3D-m1 |
| SizeZoneNonUniformityNormalized | glszm | lbp-3D-m1 |
| SizeZoneNonUniformity | glszm | lbp-3D-m1 |
| GrayLevelNonUniformity | glszm | lbp-3D-m1 |
| LargeAreaEmphasis | glszm | lbp-3D-m1 |
| SmallAreaHighGrayLevelEmphasis | glszm | lbp-3D-m1 |
| ZonePercentage | glszm | lbp-3D-m1 |
| LargeAreaLowGrayLevelEmphasis | glszm | lbp-3D-m1 |
| LargeAreaHighGrayLevelEmphasis | glszm | lbp-3D-m1 |
| HighGrayLevelZoneEmphasis | glszm | lbp-3D-m1 |
| SmallAreaEmphasis | glszm | lbp-3D-m1 |
| LowGrayLevelZoneEmphasis | glszm | lbp-3D-m1 |
| ZoneEntropy | glszm | lbp-3D-m1 |
| SmallAreaLowGrayLevelEmphasis | glszm | lbp-3D-m1 |
| Coarseness | ngtdm | lbp-3D-m1 |
| Complexity | ngtdm | lbp-3D-m1 |
| Strength | ngtdm | lbp-3D-m1 |
| Contrast | ngtdm | lbp-3D-m1 |
| Busyness | ngtdm | lbp-3D-m1 |
| InterquartileRange | firstorder | lbp-3D-m2 |
| Skewness | firstorder | lbp-3D-m2 |
| Uniformity | firstorder | lbp-3D-m2 |
| Median | firstorder | lbp-3D-m2 |
| Energy | firstorder | lbp-3D-m2 |
| RobustMeanAbsoluteDeviation | firstorder | lbp-3D-m2 |
| MeanAbsoluteDeviation | firstorder | lbp-3D-m2 |
| TotalEnergy | firstorder | lbp-3D-m2 |
| Maximum | firstorder | lbp-3D-m2 |
| RootMeanSquared | firstorder | lbp-3D-m2 |
| 90Percentile | firstorder | lbp-3D-m2 |
| Minimum | firstorder | lbp-3D-m2 |
| Entropy | firstorder | lbp-3D-m2 |
| Range | firstorder | lbp-3D-m2 |
| Variance | firstorder | lbp-3D-m2 |
| 10Percentile | firstorder | lbp-3D-m2 |
| Kurtosis | firstorder | lbp-3D-m2 |
| Mean | firstorder | lbp-3D-m2 |
| JointAverage | glcm | lbp-3D-m2 |
| SumAverage | glcm | lbp-3D-m2 |
| JointEntropy | glcm | lbp-3D-m2 |
| ClusterShade | glcm | lbp-3D-m2 |
| MaximumProbability | glcm | lbp-3D-m2 |
| Idmn | glcm | lbp-3D-m2 |
| JointEnergy | glcm | lbp-3D-m2 |
| Contrast | glcm | lbp-3D-m2 |
| DifferenceEntropy | glcm | lbp-3D-m2 |
| InverseVariance | glcm | lbp-3D-m2 |
| DifferenceVariance | glcm | lbp-3D-m2 |
| Idn | glcm | lbp-3D-m2 |
| Idm | glcm | lbp-3D-m2 |
| Correlation | glcm | lbp-3D-m2 |
| Autocorrelation | glcm | lbp-3D-m2 |
| SumEntropy | glcm | lbp-3D-m2 |
| MCC | glcm | lbp-3D-m2 |
| SumSquares | glcm | lbp-3D-m2 |
| ClusterProminence | glcm | lbp-3D-m2 |
| Imc2 | glcm | lbp-3D-m2 |
| Imc1 | glcm | lbp-3D-m2 |
| DifferenceAverage | glcm | lbp-3D-m2 |
| Id | glcm | lbp-3D-m2 |
| ClusterTendency | glcm | lbp-3D-m2 |
| GrayLevelVariance | gldm | lbp-3D-m2 |
| HighGrayLevelEmphasis | gldm | lbp-3D-m2 |
| DependenceEntropy | gldm | lbp-3D-m2 |
| DependenceNonUniformity | gldm | lbp-3D-m2 |
| GrayLevelNonUniformity | gldm | lbp-3D-m2 |
| SmallDependenceEmphasis | gldm | lbp-3D-m2 |
| SmallDependenceHighGrayLevelEmphasis | gldm | lbp-3D-m2 |
| DependenceNonUniformityNormalized | gldm | lbp-3D-m2 |
| LargeDependenceEmphasis | gldm | lbp-3D-m2 |
| LargeDependenceLowGrayLevelEmphasis | gldm | lbp-3D-m2 |
| DependenceVariance | gldm | lbp-3D-m2 |
| LargeDependenceHighGrayLevelEmphasis | gldm | lbp-3D-m2 |
| SmallDependenceLowGrayLevelEmphasis | gldm | lbp-3D-m2 |
| LowGrayLevelEmphasis | gldm | lbp-3D-m2 |
| ShortRunLowGrayLevelEmphasis | glrlm | lbp-3D-m2 |
| GrayLevelVariance | glrlm | lbp-3D-m2 |
| LowGrayLevelRunEmphasis | glrlm | lbp-3D-m2 |
| GrayLevelNonUniformityNormalized | glrlm | lbp-3D-m2 |
| RunVariance | glrlm | lbp-3D-m2 |
| GrayLevelNonUniformity | glrlm | lbp-3D-m2 |
| LongRunEmphasis | glrlm | lbp-3D-m2 |
| ShortRunHighGrayLevelEmphasis | glrlm | lbp-3D-m2 |
| RunLengthNonUniformity | glrlm | lbp-3D-m2 |
| ShortRunEmphasis | glrlm | lbp-3D-m2 |
| LongRunHighGrayLevelEmphasis | glrlm | lbp-3D-m2 |
| RunPercentage | glrlm | lbp-3D-m2 |
| LongRunLowGrayLevelEmphasis | glrlm | lbp-3D-m2 |
| RunEntropy | glrlm | lbp-3D-m2 |
| HighGrayLevelRunEmphasis | glrlm | lbp-3D-m2 |
| RunLengthNonUniformityNormalized | glrlm | lbp-3D-m2 |
| GrayLevelVariance | glszm | lbp-3D-m2 |
| ZoneVariance | glszm | lbp-3D-m2 |
| GrayLevelNonUniformityNormalized | glszm | lbp-3D-m2 |
| SizeZoneNonUniformityNormalized | glszm | lbp-3D-m2 |
| SizeZoneNonUniformity | glszm | lbp-3D-m2 |
| GrayLevelNonUniformity | glszm | lbp-3D-m2 |
| LargeAreaEmphasis | glszm | lbp-3D-m2 |
| SmallAreaHighGrayLevelEmphasis | glszm | lbp-3D-m2 |
| ZonePercentage | glszm | lbp-3D-m2 |
| LargeAreaLowGrayLevelEmphasis | glszm | lbp-3D-m2 |
| LargeAreaHighGrayLevelEmphasis | glszm | lbp-3D-m2 |
| HighGrayLevelZoneEmphasis | glszm | lbp-3D-m2 |
| SmallAreaEmphasis | glszm | lbp-3D-m2 |
| LowGrayLevelZoneEmphasis | glszm | lbp-3D-m2 |
| ZoneEntropy | glszm | lbp-3D-m2 |
| SmallAreaLowGrayLevelEmphasis | glszm | lbp-3D-m2 |
| Coarseness | ngtdm | lbp-3D-m2 |
| Complexity | ngtdm | lbp-3D-m2 |
| Strength | ngtdm | lbp-3D-m2 |
| Contrast | ngtdm | lbp-3D-m2 |
| Busyness | ngtdm | lbp-3D-m2 |
| InterquartileRange | firstorder | lbp-3D-k |
| Skewness | firstorder | lbp-3D-k |
| Uniformity | firstorder | lbp-3D-k |
| Median | firstorder | lbp-3D-k |
| Energy | firstorder | lbp-3D-k |
| RobustMeanAbsoluteDeviation | firstorder | lbp-3D-k |
| MeanAbsoluteDeviation | firstorder | lbp-3D-k |
| TotalEnergy | firstorder | lbp-3D-k |
| Maximum | firstorder | lbp-3D-k |
| RootMeanSquared | firstorder | lbp-3D-k |
| 90Percentile | firstorder | lbp-3D-k |
| Minimum | firstorder | lbp-3D-k |
| Entropy | firstorder | lbp-3D-k |
| Range | firstorder | lbp-3D-k |
| Variance | firstorder | lbp-3D-k |
| 10Percentile | firstorder | lbp-3D-k |
| Kurtosis | firstorder | lbp-3D-k |
| Mean | firstorder | lbp-3D-k |
| JointAverage | glcm | lbp-3D-k |
| SumAverage | glcm | lbp-3D-k |
| JointEntropy | glcm | lbp-3D-k |
| ClusterShade | glcm | lbp-3D-k |
| MaximumProbability | glcm | lbp-3D-k |
| Idmn | glcm | lbp-3D-k |
| JointEnergy | glcm | lbp-3D-k |
| Contrast | glcm | lbp-3D-k |
| DifferenceEntropy | glcm | lbp-3D-k |
| InverseVariance | glcm | lbp-3D-k |
| DifferenceVariance | glcm | lbp-3D-k |
| Idn | glcm | lbp-3D-k |
| Idm | glcm | lbp-3D-k |
| Correlation | glcm | lbp-3D-k |
| Autocorrelation | glcm | lbp-3D-k |
| SumEntropy | glcm | lbp-3D-k |
| MCC | glcm | lbp-3D-k |
| SumSquares | glcm | lbp-3D-k |
| ClusterProminence | glcm | lbp-3D-k |
| Imc2 | glcm | lbp-3D-k |
| Imc1 | glcm | lbp-3D-k |
| DifferenceAverage | glcm | lbp-3D-k |
| Id | glcm | lbp-3D-k |
| ClusterTendency | glcm | lbp-3D-k |
| GrayLevelVariance | gldm | lbp-3D-k |
| HighGrayLevelEmphasis | gldm | lbp-3D-k |
| DependenceEntropy | gldm | lbp-3D-k |
| DependenceNonUniformity | gldm | lbp-3D-k |
| GrayLevelNonUniformity | gldm | lbp-3D-k |
| SmallDependenceEmphasis | gldm | lbp-3D-k |
| SmallDependenceHighGrayLevelEmphasis | gldm | lbp-3D-k |
| DependenceNonUniformityNormalized | gldm | lbp-3D-k |
| LargeDependenceEmphasis | gldm | lbp-3D-k |
| LargeDependenceLowGrayLevelEmphasis | gldm | lbp-3D-k |
| DependenceVariance | gldm | lbp-3D-k |
| LargeDependenceHighGrayLevelEmphasis | gldm | lbp-3D-k |
| SmallDependenceLowGrayLevelEmphasis | gldm | lbp-3D-k |
| LowGrayLevelEmphasis | gldm | lbp-3D-k |
| ShortRunLowGrayLevelEmphasis | glrlm | lbp-3D-k |
| GrayLevelVariance | glrlm | lbp-3D-k |
| LowGrayLevelRunEmphasis | glrlm | lbp-3D-k |
| GrayLevelNonUniformityNormalized | glrlm | lbp-3D-k |
| RunVariance | glrlm | lbp-3D-k |
| GrayLevelNonUniformity | glrlm | lbp-3D-k |
| LongRunEmphasis | glrlm | lbp-3D-k |
| ShortRunHighGrayLevelEmphasis | glrlm | lbp-3D-k |
| RunLengthNonUniformity | glrlm | lbp-3D-k |
| ShortRunEmphasis | glrlm | lbp-3D-k |
| LongRunHighGrayLevelEmphasis | glrlm | lbp-3D-k |
| RunPercentage | glrlm | lbp-3D-k |
| LongRunLowGrayLevelEmphasis | glrlm | lbp-3D-k |
| RunEntropy | glrlm | lbp-3D-k |
| HighGrayLevelRunEmphasis | glrlm | lbp-3D-k |
| RunLengthNonUniformityNormalized | glrlm | lbp-3D-k |
| GrayLevelVariance | glszm | lbp-3D-k |
| ZoneVariance | glszm | lbp-3D-k |
| GrayLevelNonUniformityNormalized | glszm | lbp-3D-k |
| SizeZoneNonUniformityNormalized | glszm | lbp-3D-k |
| SizeZoneNonUniformity | glszm | lbp-3D-k |
| GrayLevelNonUniformity | glszm | lbp-3D-k |
| LargeAreaEmphasis | glszm | lbp-3D-k |
| SmallAreaHighGrayLevelEmphasis | glszm | lbp-3D-k |
| ZonePercentage | glszm | lbp-3D-k |
| LargeAreaLowGrayLevelEmphasis | glszm | lbp-3D-k |
| LargeAreaHighGrayLevelEmphasis | glszm | lbp-3D-k |
| HighGrayLevelZoneEmphasis | glszm | lbp-3D-k |
| SmallAreaEmphasis | glszm | lbp-3D-k |
| LowGrayLevelZoneEmphasis | glszm | lbp-3D-k |
| ZoneEntropy | glszm | lbp-3D-k |
| SmallAreaLowGrayLevelEmphasis | glszm | lbp-3D-k |
| Coarseness | ngtdm | lbp-3D-k |
| Complexity | ngtdm | lbp-3D-k |
| Strength | ngtdm | lbp-3D-k |
| Contrast | ngtdm | lbp-3D-k |
| Busyness | ngtdm | lbp-3D-k |
| InterquartileRange | firstorder | wavelet-LHL |
| Skewness | firstorder | wavelet-LHL |
| Uniformity | firstorder | wavelet-LHL |
| Median | firstorder | wavelet-LHL |
| Energy | firstorder | wavelet-LHL |
| RobustMeanAbsoluteDeviation | firstorder | wavelet-LHL |
| MeanAbsoluteDeviation | firstorder | wavelet-LHL |
| TotalEnergy | firstorder | wavelet-LHL |
| Maximum | firstorder | wavelet-LHL |
| RootMeanSquared | firstorder | wavelet-LHL |
| 90Percentile | firstorder | wavelet-LHL |
| Minimum | firstorder | wavelet-LHL |
| Entropy | firstorder | wavelet-LHL |
| Range | firstorder | wavelet-LHL |
| Variance | firstorder | wavelet-LHL |
| 10Percentile | firstorder | wavelet-LHL |
| Kurtosis | firstorder | wavelet-LHL |
| Mean | firstorder | wavelet-LHL |
| JointAverage | glcm | wavelet-LHL |
| SumAverage | glcm | wavelet-LHL |
| JointEntropy | glcm | wavelet-LHL |
| ClusterShade | glcm | wavelet-LHL |
| MaximumProbability | glcm | wavelet-LHL |
| Idmn | glcm | wavelet-LHL |
| JointEnergy | glcm | wavelet-LHL |
| Contrast | glcm | wavelet-LHL |
| DifferenceEntropy | glcm | wavelet-LHL |
| InverseVariance | glcm | wavelet-LHL |
| DifferenceVariance | glcm | wavelet-LHL |
| Idn | glcm | wavelet-LHL |
| Idm | glcm | wavelet-LHL |
| Correlation | glcm | wavelet-LHL |
| Autocorrelation | glcm | wavelet-LHL |
| SumEntropy | glcm | wavelet-LHL |
| MCC | glcm | wavelet-LHL |
| SumSquares | glcm | wavelet-LHL |
| ClusterProminence | glcm | wavelet-LHL |
| Imc2 | glcm | wavelet-LHL |
| Imc1 | glcm | wavelet-LHL |
| DifferenceAverage | glcm | wavelet-LHL |
| Id | glcm | wavelet-LHL |
| ClusterTendency | glcm | wavelet-LHL |
| GrayLevelVariance | gldm | wavelet-LHL |
| HighGrayLevelEmphasis | gldm | wavelet-LHL |
| DependenceEntropy | gldm | wavelet-LHL |
| DependenceNonUniformity | gldm | wavelet-LHL |
| GrayLevelNonUniformity | gldm | wavelet-LHL |
| SmallDependenceEmphasis | gldm | wavelet-LHL |
| SmallDependenceHighGrayLevelEmphasis | gldm | wavelet-LHL |
| DependenceNonUniformityNormalized | gldm | wavelet-LHL |
| LargeDependenceEmphasis | gldm | wavelet-LHL |
| LargeDependenceLowGrayLevelEmphasis | gldm | wavelet-LHL |
| DependenceVariance | gldm | wavelet-LHL |
| LargeDependenceHighGrayLevelEmphasis | gldm | wavelet-LHL |
| SmallDependenceLowGrayLevelEmphasis | gldm | wavelet-LHL |
| LowGrayLevelEmphasis | gldm | wavelet-LHL |
| ShortRunLowGrayLevelEmphasis | glrlm | wavelet-LHL |
| GrayLevelVariance | glrlm | wavelet-LHL |
| LowGrayLevelRunEmphasis | glrlm | wavelet-LHL |
| GrayLevelNonUniformityNormalized | glrlm | wavelet-LHL |
| RunVariance | glrlm | wavelet-LHL |
| GrayLevelNonUniformity | glrlm | wavelet-LHL |
| LongRunEmphasis | glrlm | wavelet-LHL |
| ShortRunHighGrayLevelEmphasis | glrlm | wavelet-LHL |
| RunLengthNonUniformity | glrlm | wavelet-LHL |
| ShortRunEmphasis | glrlm | wavelet-LHL |
| LongRunHighGrayLevelEmphasis | glrlm | wavelet-LHL |
| RunPercentage | glrlm | wavelet-LHL |
| LongRunLowGrayLevelEmphasis | glrlm | wavelet-LHL |
| RunEntropy | glrlm | wavelet-LHL |
| HighGrayLevelRunEmphasis | glrlm | wavelet-LHL |
| RunLengthNonUniformityNormalized | glrlm | wavelet-LHL |
| GrayLevelVariance | glszm | wavelet-LHL |
| ZoneVariance | glszm | wavelet-LHL |
| GrayLevelNonUniformityNormalized | glszm | wavelet-LHL |
| SizeZoneNonUniformityNormalized | glszm | wavelet-LHL |
| SizeZoneNonUniformity | glszm | wavelet-LHL |
| GrayLevelNonUniformity | glszm | wavelet-LHL |
| LargeAreaEmphasis | glszm | wavelet-LHL |
| SmallAreaHighGrayLevelEmphasis | glszm | wavelet-LHL |
| ZonePercentage | glszm | wavelet-LHL |
| LargeAreaLowGrayLevelEmphasis | glszm | wavelet-LHL |
| LargeAreaHighGrayLevelEmphasis | glszm | wavelet-LHL |
| HighGrayLevelZoneEmphasis | glszm | wavelet-LHL |
| SmallAreaEmphasis | glszm | wavelet-LHL |
| LowGrayLevelZoneEmphasis | glszm | wavelet-LHL |
| ZoneEntropy | glszm | wavelet-LHL |
| SmallAreaLowGrayLevelEmphasis | glszm | wavelet-LHL |
| Coarseness | ngtdm | wavelet-LHL |
| Complexity | ngtdm | wavelet-LHL |
| Strength | ngtdm | wavelet-LHL |
| Contrast | ngtdm | wavelet-LHL |
| Busyness | ngtdm | wavelet-LHL |
| InterquartileRange | firstorder | wavelet-LHH |
| Skewness | firstorder | wavelet-LHH |
| Uniformity | firstorder | wavelet-LHH |
| Median | firstorder | wavelet-LHH |
| Energy | firstorder | wavelet-LHH |
| RobustMeanAbsoluteDeviation | firstorder | wavelet-LHH |
| MeanAbsoluteDeviation | firstorder | wavelet-LHH |
| TotalEnergy | firstorder | wavelet-LHH |
| Maximum | firstorder | wavelet-LHH |
| RootMeanSquared | firstorder | wavelet-LHH |
| 90Percentile | firstorder | wavelet-LHH |
| Minimum | firstorder | wavelet-LHH |
| Entropy | firstorder | wavelet-LHH |
| Range | firstorder | wavelet-LHH |
| Variance | firstorder | wavelet-LHH |
| 10Percentile | firstorder | wavelet-LHH |
| Kurtosis | firstorder | wavelet-LHH |
| Mean | firstorder | wavelet-LHH |
| JointAverage | glcm | wavelet-LHH |
| SumAverage | glcm | wavelet-LHH |
| JointEntropy | glcm | wavelet-LHH |
| ClusterShade | glcm | wavelet-LHH |
| MaximumProbability | glcm | wavelet-LHH |
| Idmn | glcm | wavelet-LHH |
| JointEnergy | glcm | wavelet-LHH |
| Contrast | glcm | wavelet-LHH |
| DifferenceEntropy | glcm | wavelet-LHH |
| InverseVariance | glcm | wavelet-LHH |
| DifferenceVariance | glcm | wavelet-LHH |
| Idn | glcm | wavelet-LHH |
| Idm | glcm | wavelet-LHH |
| Correlation | glcm | wavelet-LHH |
| Autocorrelation | glcm | wavelet-LHH |
| SumEntropy | glcm | wavelet-LHH |
| MCC | glcm | wavelet-LHH |
| SumSquares | glcm | wavelet-LHH |
| ClusterProminence | glcm | wavelet-LHH |
| Imc2 | glcm | wavelet-LHH |
| Imc1 | glcm | wavelet-LHH |
| DifferenceAverage | glcm | wavelet-LHH |
| Id | glcm | wavelet-LHH |
| ClusterTendency | glcm | wavelet-LHH |
| GrayLevelVariance | gldm | wavelet-LHH |
| HighGrayLevelEmphasis | gldm | wavelet-LHH |
| DependenceEntropy | gldm | wavelet-LHH |
| DependenceNonUniformity | gldm | wavelet-LHH |
| GrayLevelNonUniformity | gldm | wavelet-LHH |
| SmallDependenceEmphasis | gldm | wavelet-LHH |
| SmallDependenceHighGrayLevelEmphasis | gldm | wavelet-LHH |
| DependenceNonUniformityNormalized | gldm | wavelet-LHH |
| LargeDependenceEmphasis | gldm | wavelet-LHH |
| LargeDependenceLowGrayLevelEmphasis | gldm | wavelet-LHH |
| DependenceVariance | gldm | wavelet-LHH |
| LargeDependenceHighGrayLevelEmphasis | gldm | wavelet-LHH |
| SmallDependenceLowGrayLevelEmphasis | gldm | wavelet-LHH |
| LowGrayLevelEmphasis | gldm | wavelet-LHH |
| ShortRunLowGrayLevelEmphasis | glrlm | wavelet-LHH |
| GrayLevelVariance | glrlm | wavelet-LHH |
| LowGrayLevelRunEmphasis | glrlm | wavelet-LHH |
| GrayLevelNonUniformityNormalized | glrlm | wavelet-LHH |
| RunVariance | glrlm | wavelet-LHH |
| GrayLevelNonUniformity | glrlm | wavelet-LHH |
| LongRunEmphasis | glrlm | wavelet-LHH |
| ShortRunHighGrayLevelEmphasis | glrlm | wavelet-LHH |
| RunLengthNonUniformity | glrlm | wavelet-LHH |
| ShortRunEmphasis | glrlm | wavelet-LHH |
| LongRunHighGrayLevelEmphasis | glrlm | wavelet-LHH |
| RunPercentage | glrlm | wavelet-LHH |
| LongRunLowGrayLevelEmphasis | glrlm | wavelet-LHH |
| RunEntropy | glrlm | wavelet-LHH |
| HighGrayLevelRunEmphasis | glrlm | wavelet-LHH |
| RunLengthNonUniformityNormalized | glrlm | wavelet-LHH |
| GrayLevelVariance | glszm | wavelet-LHH |
| ZoneVariance | glszm | wavelet-LHH |
| GrayLevelNonUniformityNormalized | glszm | wavelet-LHH |
| SizeZoneNonUniformityNormalized | glszm | wavelet-LHH |
| SizeZoneNonUniformity | glszm | wavelet-LHH |
| GrayLevelNonUniformity | glszm | wavelet-LHH |
| LargeAreaEmphasis | glszm | wavelet-LHH |
| SmallAreaHighGrayLevelEmphasis | glszm | wavelet-LHH |
| ZonePercentage | glszm | wavelet-LHH |
| LargeAreaLowGrayLevelEmphasis | glszm | wavelet-LHH |
| LargeAreaHighGrayLevelEmphasis | glszm | wavelet-LHH |
| HighGrayLevelZoneEmphasis | glszm | wavelet-LHH |
| SmallAreaEmphasis | glszm | wavelet-LHH |
| LowGrayLevelZoneEmphasis | glszm | wavelet-LHH |
| ZoneEntropy | glszm | wavelet-LHH |
| SmallAreaLowGrayLevelEmphasis | glszm | wavelet-LHH |
| Coarseness | ngtdm | wavelet-LHH |
| Complexity | ngtdm | wavelet-LHH |
| Strength | ngtdm | wavelet-LHH |
| Contrast | ngtdm | wavelet-LHH |
| Busyness | ngtdm | wavelet-LHH |
| InterquartileRange | firstorder | wavelet-HLL |
| Skewness | firstorder | wavelet-HLL |
| Uniformity | firstorder | wavelet-HLL |
| Median | firstorder | wavelet-HLL |
| Energy | firstorder | wavelet-HLL |
| RobustMeanAbsoluteDeviation | firstorder | wavelet-HLL |
| MeanAbsoluteDeviation | firstorder | wavelet-HLL |
| TotalEnergy | firstorder | wavelet-HLL |
| Maximum | firstorder | wavelet-HLL |
| RootMeanSquared | firstorder | wavelet-HLL |
| 90Percentile | firstorder | wavelet-HLL |
| Minimum | firstorder | wavelet-HLL |
| Entropy | firstorder | wavelet-HLL |
| Range | firstorder | wavelet-HLL |
| Variance | firstorder | wavelet-HLL |
| 10Percentile | firstorder | wavelet-HLL |
| Kurtosis | firstorder | wavelet-HLL |
| Mean | firstorder | wavelet-HLL |
| JointAverage | glcm | wavelet-HLL |
| SumAverage | glcm | wavelet-HLL |
| JointEntropy | glcm | wavelet-HLL |
| ClusterShade | glcm | wavelet-HLL |
| MaximumProbability | glcm | wavelet-HLL |
| Idmn | glcm | wavelet-HLL |
| JointEnergy | glcm | wavelet-HLL |
| Contrast | glcm | wavelet-HLL |
| DifferenceEntropy | glcm | wavelet-HLL |
| InverseVariance | glcm | wavelet-HLL |
| DifferenceVariance | glcm | wavelet-HLL |
| Idn | glcm | wavelet-HLL |
| Idm | glcm | wavelet-HLL |
| Correlation | glcm | wavelet-HLL |
| Autocorrelation | glcm | wavelet-HLL |
| SumEntropy | glcm | wavelet-HLL |
| MCC | glcm | wavelet-HLL |
| SumSquares | glcm | wavelet-HLL |
| ClusterProminence | glcm | wavelet-HLL |
| Imc2 | glcm | wavelet-HLL |
| Imc1 | glcm | wavelet-HLL |
| DifferenceAverage | glcm | wavelet-HLL |
| Id | glcm | wavelet-HLL |
| ClusterTendency | glcm | wavelet-HLL |
| GrayLevelVariance | gldm | wavelet-HLL |
| HighGrayLevelEmphasis | gldm | wavelet-HLL |
| DependenceEntropy | gldm | wavelet-HLL |
| DependenceNonUniformity | gldm | wavelet-HLL |
| GrayLevelNonUniformity | gldm | wavelet-HLL |
| SmallDependenceEmphasis | gldm | wavelet-HLL |
| SmallDependenceHighGrayLevelEmphasis | gldm | wavelet-HLL |
| DependenceNonUniformityNormalized | gldm | wavelet-HLL |
| LargeDependenceEmphasis | gldm | wavelet-HLL |
| LargeDependenceLowGrayLevelEmphasis | gldm | wavelet-HLL |
| DependenceVariance | gldm | wavelet-HLL |
| LargeDependenceHighGrayLevelEmphasis | gldm | wavelet-HLL |
| SmallDependenceLowGrayLevelEmphasis | gldm | wavelet-HLL |
| LowGrayLevelEmphasis | gldm | wavelet-HLL |
| ShortRunLowGrayLevelEmphasis | glrlm | wavelet-HLL |
| GrayLevelVariance | glrlm | wavelet-HLL |
| LowGrayLevelRunEmphasis | glrlm | wavelet-HLL |
| GrayLevelNonUniformityNormalized | glrlm | wavelet-HLL |
| RunVariance | glrlm | wavelet-HLL |
| GrayLevelNonUniformity | glrlm | wavelet-HLL |
| LongRunEmphasis | glrlm | wavelet-HLL |
| ShortRunHighGrayLevelEmphasis | glrlm | wavelet-HLL |
| RunLengthNonUniformity | glrlm | wavelet-HLL |
| ShortRunEmphasis | glrlm | wavelet-HLL |
| LongRunHighGrayLevelEmphasis | glrlm | wavelet-HLL |
| RunPercentage | glrlm | wavelet-HLL |
| LongRunLowGrayLevelEmphasis | glrlm | wavelet-HLL |
| RunEntropy | glrlm | wavelet-HLL |
| HighGrayLevelRunEmphasis | glrlm | wavelet-HLL |
| RunLengthNonUniformityNormalized | glrlm | wavelet-HLL |
| GrayLevelVariance | glszm | wavelet-HLL |
| ZoneVariance | glszm | wavelet-HLL |
| GrayLevelNonUniformityNormalized | glszm | wavelet-HLL |
| SizeZoneNonUniformityNormalized | glszm | wavelet-HLL |
| SizeZoneNonUniformity | glszm | wavelet-HLL |
| GrayLevelNonUniformity | glszm | wavelet-HLL |
| LargeAreaEmphasis | glszm | wavelet-HLL |
| SmallAreaHighGrayLevelEmphasis | glszm | wavelet-HLL |
| ZonePercentage | glszm | wavelet-HLL |
| LargeAreaLowGrayLevelEmphasis | glszm | wavelet-HLL |
| LargeAreaHighGrayLevelEmphasis | glszm | wavelet-HLL |
| HighGrayLevelZoneEmphasis | glszm | wavelet-HLL |
| SmallAreaEmphasis | glszm | wavelet-HLL |
| LowGrayLevelZoneEmphasis | glszm | wavelet-HLL |
| ZoneEntropy | glszm | wavelet-HLL |
| SmallAreaLowGrayLevelEmphasis | glszm | wavelet-HLL |
| Coarseness | ngtdm | wavelet-HLL |
| Complexity | ngtdm | wavelet-HLL |
| Strength | ngtdm | wavelet-HLL |
| Contrast | ngtdm | wavelet-HLL |
| Busyness | ngtdm | wavelet-HLL |
| InterquartileRange | firstorder | wavelet-LLH |
| Skewness | firstorder | wavelet-LLH |
| Uniformity | firstorder | wavelet-LLH |
| Median | firstorder | wavelet-LLH |
| Energy | firstorder | wavelet-LLH |
| RobustMeanAbsoluteDeviation | firstorder | wavelet-LLH |
| MeanAbsoluteDeviation | firstorder | wavelet-LLH |
| TotalEnergy | firstorder | wavelet-LLH |
| Maximum | firstorder | wavelet-LLH |
| RootMeanSquared | firstorder | wavelet-LLH |
| 90Percentile | firstorder | wavelet-LLH |
| Minimum | firstorder | wavelet-LLH |
| Entropy | firstorder | wavelet-LLH |
| Range | firstorder | wavelet-LLH |
| Variance | firstorder | wavelet-LLH |
| 10Percentile | firstorder | wavelet-LLH |
| Kurtosis | firstorder | wavelet-LLH |
| Mean | firstorder | wavelet-LLH |
| JointAverage | glcm | wavelet-LLH |
| SumAverage | glcm | wavelet-LLH |
| JointEntropy | glcm | wavelet-LLH |
| ClusterShade | glcm | wavelet-LLH |
| MaximumProbability | glcm | wavelet-LLH |
| Idmn | glcm | wavelet-LLH |
| JointEnergy | glcm | wavelet-LLH |
| Contrast | glcm | wavelet-LLH |
| DifferenceEntropy | glcm | wavelet-LLH |
| InverseVariance | glcm | wavelet-LLH |
| DifferenceVariance | glcm | wavelet-LLH |
| Idn | glcm | wavelet-LLH |
| Idm | glcm | wavelet-LLH |
| Correlation | glcm | wavelet-LLH |
| Autocorrelation | glcm | wavelet-LLH |
| SumEntropy | glcm | wavelet-LLH |
| MCC | glcm | wavelet-LLH |
| SumSquares | glcm | wavelet-LLH |
| ClusterProminence | glcm | wavelet-LLH |
| Imc2 | glcm | wavelet-LLH |
| Imc1 | glcm | wavelet-LLH |
| DifferenceAverage | glcm | wavelet-LLH |
| Id | glcm | wavelet-LLH |
| ClusterTendency | glcm | wavelet-LLH |
| GrayLevelVariance | gldm | wavelet-LLH |
| HighGrayLevelEmphasis | gldm | wavelet-LLH |
| DependenceEntropy | gldm | wavelet-LLH |
| DependenceNonUniformity | gldm | wavelet-LLH |
| GrayLevelNonUniformity | gldm | wavelet-LLH |
| SmallDependenceEmphasis | gldm | wavelet-LLH |
| SmallDependenceHighGrayLevelEmphasis | gldm | wavelet-LLH |
| DependenceNonUniformityNormalized | gldm | wavelet-LLH |
| LargeDependenceEmphasis | gldm | wavelet-LLH |
| LargeDependenceLowGrayLevelEmphasis | gldm | wavelet-LLH |
| DependenceVariance | gldm | wavelet-LLH |
| LargeDependenceHighGrayLevelEmphasis | gldm | wavelet-LLH |
| SmallDependenceLowGrayLevelEmphasis | gldm | wavelet-LLH |
| LowGrayLevelEmphasis | gldm | wavelet-LLH |
| ShortRunLowGrayLevelEmphasis | glrlm | wavelet-LLH |
| GrayLevelVariance | glrlm | wavelet-LLH |
| LowGrayLevelRunEmphasis | glrlm | wavelet-LLH |
| GrayLevelNonUniformityNormalized | glrlm | wavelet-LLH |
| RunVariance | glrlm | wavelet-LLH |
| GrayLevelNonUniformity | glrlm | wavelet-LLH |
| LongRunEmphasis | glrlm | wavelet-LLH |
| ShortRunHighGrayLevelEmphasis | glrlm | wavelet-LLH |
| RunLengthNonUniformity | glrlm | wavelet-LLH |
| ShortRunEmphasis | glrlm | wavelet-LLH |
| LongRunHighGrayLevelEmphasis | glrlm | wavelet-LLH |
| RunPercentage | glrlm | wavelet-LLH |
| LongRunLowGrayLevelEmphasis | glrlm | wavelet-LLH |
| RunEntropy | glrlm | wavelet-LLH |
| HighGrayLevelRunEmphasis | glrlm | wavelet-LLH |
| RunLengthNonUniformityNormalized | glrlm | wavelet-LLH |
| GrayLevelVariance | glszm | wavelet-LLH |
| ZoneVariance | glszm | wavelet-LLH |
| GrayLevelNonUniformityNormalized | glszm | wavelet-LLH |
| SizeZoneNonUniformityNormalized | glszm | wavelet-LLH |
| SizeZoneNonUniformity | glszm | wavelet-LLH |
| GrayLevelNonUniformity | glszm | wavelet-LLH |
| LargeAreaEmphasis | glszm | wavelet-LLH |
| SmallAreaHighGrayLevelEmphasis | glszm | wavelet-LLH |
| ZonePercentage | glszm | wavelet-LLH |
| LargeAreaLowGrayLevelEmphasis | glszm | wavelet-LLH |
| LargeAreaHighGrayLevelEmphasis | glszm | wavelet-LLH |
| HighGrayLevelZoneEmphasis | glszm | wavelet-LLH |
| SmallAreaEmphasis | glszm | wavelet-LLH |
| LowGrayLevelZoneEmphasis | glszm | wavelet-LLH |
| ZoneEntropy | glszm | wavelet-LLH |
| SmallAreaLowGrayLevelEmphasis | glszm | wavelet-LLH |
| Coarseness | ngtdm | wavelet-LLH |
| Complexity | ngtdm | wavelet-LLH |
| Strength | ngtdm | wavelet-LLH |
| Contrast | ngtdm | wavelet-LLH |
| Busyness | ngtdm | wavelet-LLH |
| InterquartileRange | firstorder | wavelet-HLH |
| Skewness | firstorder | wavelet-HLH |
| Uniformity | firstorder | wavelet-HLH |
| Median | firstorder | wavelet-HLH |
| Energy | firstorder | wavelet-HLH |
| RobustMeanAbsoluteDeviation | firstorder | wavelet-HLH |
| MeanAbsoluteDeviation | firstorder | wavelet-HLH |
| TotalEnergy | firstorder | wavelet-HLH |
| Maximum | firstorder | wavelet-HLH |
| RootMeanSquared | firstorder | wavelet-HLH |
| 90Percentile | firstorder | wavelet-HLH |
| Minimum | firstorder | wavelet-HLH |
| Entropy | firstorder | wavelet-HLH |
| Range | firstorder | wavelet-HLH |
| Variance | firstorder | wavelet-HLH |
| 10Percentile | firstorder | wavelet-HLH |
| Kurtosis | firstorder | wavelet-HLH |
| Mean | firstorder | wavelet-HLH |
| JointAverage | glcm | wavelet-HLH |
| SumAverage | glcm | wavelet-HLH |
| JointEntropy | glcm | wavelet-HLH |
| ClusterShade | glcm | wavelet-HLH |
| MaximumProbability | glcm | wavelet-HLH |
| Idmn | glcm | wavelet-HLH |
| JointEnergy | glcm | wavelet-HLH |
| Contrast | glcm | wavelet-HLH |
| DifferenceEntropy | glcm | wavelet-HLH |
| InverseVariance | glcm | wavelet-HLH |
| DifferenceVariance | glcm | wavelet-HLH |
| Idn | glcm | wavelet-HLH |
| Idm | glcm | wavelet-HLH |
| Correlation | glcm | wavelet-HLH |
| Autocorrelation | glcm | wavelet-HLH |
| SumEntropy | glcm | wavelet-HLH |
| MCC | glcm | wavelet-HLH |
| SumSquares | glcm | wavelet-HLH |
| ClusterProminence | glcm | wavelet-HLH |
| Imc2 | glcm | wavelet-HLH |
| Imc1 | glcm | wavelet-HLH |
| DifferenceAverage | glcm | wavelet-HLH |
| Id | glcm | wavelet-HLH |
| ClusterTendency | glcm | wavelet-HLH |
| GrayLevelVariance | gldm | wavelet-HLH |
| HighGrayLevelEmphasis | gldm | wavelet-HLH |
| DependenceEntropy | gldm | wavelet-HLH |
| DependenceNonUniformity | gldm | wavelet-HLH |
| GrayLevelNonUniformity | gldm | wavelet-HLH |
| SmallDependenceEmphasis | gldm | wavelet-HLH |
| SmallDependenceHighGrayLevelEmphasis | gldm | wavelet-HLH |
| DependenceNonUniformityNormalized | gldm | wavelet-HLH |
| LargeDependenceEmphasis | gldm | wavelet-HLH |
| LargeDependenceLowGrayLevelEmphasis | gldm | wavelet-HLH |
| DependenceVariance | gldm | wavelet-HLH |
| LargeDependenceHighGrayLevelEmphasis | gldm | wavelet-HLH |
| SmallDependenceLowGrayLevelEmphasis | gldm | wavelet-HLH |
| LowGrayLevelEmphasis | gldm | wavelet-HLH |
| ShortRunLowGrayLevelEmphasis | glrlm | wavelet-HLH |
| GrayLevelVariance | glrlm | wavelet-HLH |
| LowGrayLevelRunEmphasis | glrlm | wavelet-HLH |
| GrayLevelNonUniformityNormalized | glrlm | wavelet-HLH |
| RunVariance | glrlm | wavelet-HLH |
| GrayLevelNonUniformity | glrlm | wavelet-HLH |
| LongRunEmphasis | glrlm | wavelet-HLH |
| ShortRunHighGrayLevelEmphasis | glrlm | wavelet-HLH |
| RunLengthNonUniformity | glrlm | wavelet-HLH |
| ShortRunEmphasis | glrlm | wavelet-HLH |
| LongRunHighGrayLevelEmphasis | glrlm | wavelet-HLH |
| RunPercentage | glrlm | wavelet-HLH |
| LongRunLowGrayLevelEmphasis | glrlm | wavelet-HLH |
| RunEntropy | glrlm | wavelet-HLH |
| HighGrayLevelRunEmphasis | glrlm | wavelet-HLH |
| RunLengthNonUniformityNormalized | glrlm | wavelet-HLH |
| GrayLevelVariance | glszm | wavelet-HLH |
| ZoneVariance | glszm | wavelet-HLH |
| GrayLevelNonUniformityNormalized | glszm | wavelet-HLH |
| SizeZoneNonUniformityNormalized | glszm | wavelet-HLH |
| SizeZoneNonUniformity | glszm | wavelet-HLH |
| GrayLevelNonUniformity | glszm | wavelet-HLH |
| LargeAreaEmphasis | glszm | wavelet-HLH |
| SmallAreaHighGrayLevelEmphasis | glszm | wavelet-HLH |
| ZonePercentage | glszm | wavelet-HLH |
| LargeAreaLowGrayLevelEmphasis | glszm | wavelet-HLH |
| LargeAreaHighGrayLevelEmphasis | glszm | wavelet-HLH |
| HighGrayLevelZoneEmphasis | glszm | wavelet-HLH |
| SmallAreaEmphasis | glszm | wavelet-HLH |
| LowGrayLevelZoneEmphasis | glszm | wavelet-HLH |
| ZoneEntropy | glszm | wavelet-HLH |
| SmallAreaLowGrayLevelEmphasis | glszm | wavelet-HLH |
| Coarseness | ngtdm | wavelet-HLH |
| Complexity | ngtdm | wavelet-HLH |
| Strength | ngtdm | wavelet-HLH |
| Contrast | ngtdm | wavelet-HLH |
| Busyness | ngtdm | wavelet-HLH |
| InterquartileRange | firstorder | wavelet-HHH |
| Skewness | firstorder | wavelet-HHH |
| Uniformity | firstorder | wavelet-HHH |
| Median | firstorder | wavelet-HHH |
| Energy | firstorder | wavelet-HHH |
| RobustMeanAbsoluteDeviation | firstorder | wavelet-HHH |
| MeanAbsoluteDeviation | firstorder | wavelet-HHH |
| TotalEnergy | firstorder | wavelet-HHH |
| Maximum | firstorder | wavelet-HHH |
| RootMeanSquared | firstorder | wavelet-HHH |
| 90Percentile | firstorder | wavelet-HHH |
| Minimum | firstorder | wavelet-HHH |
| Entropy | firstorder | wavelet-HHH |
| Range | firstorder | wavelet-HHH |
| Variance | firstorder | wavelet-HHH |
| 10Percentile | firstorder | wavelet-HHH |
| Kurtosis | firstorder | wavelet-HHH |
| Mean | firstorder | wavelet-HHH |
| JointAverage | glcm | wavelet-HHH |
| SumAverage | glcm | wavelet-HHH |
| JointEntropy | glcm | wavelet-HHH |
| ClusterShade | glcm | wavelet-HHH |
| MaximumProbability | glcm | wavelet-HHH |
| Idmn | glcm | wavelet-HHH |
| JointEnergy | glcm | wavelet-HHH |
| Contrast | glcm | wavelet-HHH |
| DifferenceEntropy | glcm | wavelet-HHH |
| InverseVariance | glcm | wavelet-HHH |
| DifferenceVariance | glcm | wavelet-HHH |
| Idn | glcm | wavelet-HHH |
| Idm | glcm | wavelet-HHH |
| Correlation | glcm | wavelet-HHH |
| Autocorrelation | glcm | wavelet-HHH |
| SumEntropy | glcm | wavelet-HHH |
| MCC | glcm | wavelet-HHH |
| SumSquares | glcm | wavelet-HHH |
| ClusterProminence | glcm | wavelet-HHH |
| Imc2 | glcm | wavelet-HHH |
| Imc1 | glcm | wavelet-HHH |
| DifferenceAverage | glcm | wavelet-HHH |
| Id | glcm | wavelet-HHH |
| ClusterTendency | glcm | wavelet-HHH |
| GrayLevelVariance | gldm | wavelet-HHH |
| HighGrayLevelEmphasis | gldm | wavelet-HHH |
| DependenceEntropy | gldm | wavelet-HHH |
| DependenceNonUniformity | gldm | wavelet-HHH |
| GrayLevelNonUniformity | gldm | wavelet-HHH |
| SmallDependenceEmphasis | gldm | wavelet-HHH |
| SmallDependenceHighGrayLevelEmphasis | gldm | wavelet-HHH |
| DependenceNonUniformityNormalized | gldm | wavelet-HHH |
| LargeDependenceEmphasis | gldm | wavelet-HHH |
| LargeDependenceLowGrayLevelEmphasis | gldm | wavelet-HHH |
| DependenceVariance | gldm | wavelet-HHH |
| LargeDependenceHighGrayLevelEmphasis | gldm | wavelet-HHH |
| SmallDependenceLowGrayLevelEmphasis | gldm | wavelet-HHH |
| LowGrayLevelEmphasis | gldm | wavelet-HHH |
| ShortRunLowGrayLevelEmphasis | glrlm | wavelet-HHH |
| GrayLevelVariance | glrlm | wavelet-HHH |
| LowGrayLevelRunEmphasis | glrlm | wavelet-HHH |
| GrayLevelNonUniformityNormalized | glrlm | wavelet-HHH |
| RunVariance | glrlm | wavelet-HHH |
| GrayLevelNonUniformity | glrlm | wavelet-HHH |
| LongRunEmphasis | glrlm | wavelet-HHH |
| ShortRunHighGrayLevelEmphasis | glrlm | wavelet-HHH |
| RunLengthNonUniformity | glrlm | wavelet-HHH |
| ShortRunEmphasis | glrlm | wavelet-HHH |
| LongRunHighGrayLevelEmphasis | glrlm | wavelet-HHH |
| RunPercentage | glrlm | wavelet-HHH |
| LongRunLowGrayLevelEmphasis | glrlm | wavelet-HHH |
| RunEntropy | glrlm | wavelet-HHH |
| HighGrayLevelRunEmphasis | glrlm | wavelet-HHH |
| RunLengthNonUniformityNormalized | glrlm | wavelet-HHH |
| GrayLevelVariance | glszm | wavelet-HHH |
| ZoneVariance | glszm | wavelet-HHH |
| GrayLevelNonUniformityNormalized | glszm | wavelet-HHH |
| SizeZoneNonUniformityNormalized | glszm | wavelet-HHH |
| SizeZoneNonUniformity | glszm | wavelet-HHH |
| GrayLevelNonUniformity | glszm | wavelet-HHH |
| LargeAreaEmphasis | glszm | wavelet-HHH |
| SmallAreaHighGrayLevelEmphasis | glszm | wavelet-HHH |
| ZonePercentage | glszm | wavelet-HHH |
| LargeAreaLowGrayLevelEmphasis | glszm | wavelet-HHH |
| LargeAreaHighGrayLevelEmphasis | glszm | wavelet-HHH |
| HighGrayLevelZoneEmphasis | glszm | wavelet-HHH |
| SmallAreaEmphasis | glszm | wavelet-HHH |
| LowGrayLevelZoneEmphasis | glszm | wavelet-HHH |
| ZoneEntropy | glszm | wavelet-HHH |
| SmallAreaLowGrayLevelEmphasis | glszm | wavelet-HHH |
| Coarseness | ngtdm | wavelet-HHH |
| Complexity | ngtdm | wavelet-HHH |
| Strength | ngtdm | wavelet-HHH |
| Contrast | ngtdm | wavelet-HHH |
| Busyness | ngtdm | wavelet-HHH |
| InterquartileRange | firstorder | wavelet-HHL |
| Skewness | firstorder | wavelet-HHL |
| Uniformity | firstorder | wavelet-HHL |
| Median | firstorder | wavelet-HHL |
| Energy | firstorder | wavelet-HHL |
| RobustMeanAbsoluteDeviation | firstorder | wavelet-HHL |
| MeanAbsoluteDeviation | firstorder | wavelet-HHL |
| TotalEnergy | firstorder | wavelet-HHL |
| Maximum | firstorder | wavelet-HHL |
| RootMeanSquared | firstorder | wavelet-HHL |
| 90Percentile | firstorder | wavelet-HHL |
| Minimum | firstorder | wavelet-HHL |
| Entropy | firstorder | wavelet-HHL |
| Range | firstorder | wavelet-HHL |
| Variance | firstorder | wavelet-HHL |
| 10Percentile | firstorder | wavelet-HHL |
| Kurtosis | firstorder | wavelet-HHL |
| Mean | firstorder | wavelet-HHL |
| JointAverage | glcm | wavelet-HHL |
| SumAverage | glcm | wavelet-HHL |
| JointEntropy | glcm | wavelet-HHL |
| ClusterShade | glcm | wavelet-HHL |
| MaximumProbability | glcm | wavelet-HHL |
| Idmn | glcm | wavelet-HHL |
| JointEnergy | glcm | wavelet-HHL |
| Contrast | glcm | wavelet-HHL |
| DifferenceEntropy | glcm | wavelet-HHL |
| InverseVariance | glcm | wavelet-HHL |
| DifferenceVariance | glcm | wavelet-HHL |
| Idn | glcm | wavelet-HHL |
| Idm | glcm | wavelet-HHL |
| Correlation | glcm | wavelet-HHL |
| Autocorrelation | glcm | wavelet-HHL |
| SumEntropy | glcm | wavelet-HHL |
| MCC | glcm | wavelet-HHL |
| SumSquares | glcm | wavelet-HHL |
| ClusterProminence | glcm | wavelet-HHL |
| Imc2 | glcm | wavelet-HHL |
| Imc1 | glcm | wavelet-HHL |
| DifferenceAverage | glcm | wavelet-HHL |
| Id | glcm | wavelet-HHL |
| ClusterTendency | glcm | wavelet-HHL |
| GrayLevelVariance | gldm | wavelet-HHL |
| HighGrayLevelEmphasis | gldm | wavelet-HHL |
| DependenceEntropy | gldm | wavelet-HHL |
| DependenceNonUniformity | gldm | wavelet-HHL |
| GrayLevelNonUniformity | gldm | wavelet-HHL |
| SmallDependenceEmphasis | gldm | wavelet-HHL |
| SmallDependenceHighGrayLevelEmphasis | gldm | wavelet-HHL |
| DependenceNonUniformityNormalized | gldm | wavelet-HHL |
| LargeDependenceEmphasis | gldm | wavelet-HHL |
| LargeDependenceLowGrayLevelEmphasis | gldm | wavelet-HHL |
| DependenceVariance | gldm | wavelet-HHL |
| LargeDependenceHighGrayLevelEmphasis | gldm | wavelet-HHL |
| SmallDependenceLowGrayLevelEmphasis | gldm | wavelet-HHL |
| LowGrayLevelEmphasis | gldm | wavelet-HHL |
| ShortRunLowGrayLevelEmphasis | glrlm | wavelet-HHL |
| GrayLevelVariance | glrlm | wavelet-HHL |
| LowGrayLevelRunEmphasis | glrlm | wavelet-HHL |
| GrayLevelNonUniformityNormalized | glrlm | wavelet-HHL |
| RunVariance | glrlm | wavelet-HHL |
| GrayLevelNonUniformity | glrlm | wavelet-HHL |
| LongRunEmphasis | glrlm | wavelet-HHL |
| ShortRunHighGrayLevelEmphasis | glrlm | wavelet-HHL |
| RunLengthNonUniformity | glrlm | wavelet-HHL |
| ShortRunEmphasis | glrlm | wavelet-HHL |
| LongRunHighGrayLevelEmphasis | glrlm | wavelet-HHL |
| RunPercentage | glrlm | wavelet-HHL |
| LongRunLowGrayLevelEmphasis | glrlm | wavelet-HHL |
| RunEntropy | glrlm | wavelet-HHL |
| HighGrayLevelRunEmphasis | glrlm | wavelet-HHL |
| RunLengthNonUniformityNormalized | glrlm | wavelet-HHL |
| GrayLevelVariance | glszm | wavelet-HHL |
| ZoneVariance | glszm | wavelet-HHL |
| GrayLevelNonUniformityNormalized | glszm | wavelet-HHL |
| SizeZoneNonUniformityNormalized | glszm | wavelet-HHL |
| SizeZoneNonUniformity | glszm | wavelet-HHL |
| GrayLevelNonUniformity | glszm | wavelet-HHL |
| LargeAreaEmphasis | glszm | wavelet-HHL |
| SmallAreaHighGrayLevelEmphasis | glszm | wavelet-HHL |
| ZonePercentage | glszm | wavelet-HHL |
| LargeAreaLowGrayLevelEmphasis | glszm | wavelet-HHL |
| LargeAreaHighGrayLevelEmphasis | glszm | wavelet-HHL |
| HighGrayLevelZoneEmphasis | glszm | wavelet-HHL |
| SmallAreaEmphasis | glszm | wavelet-HHL |
| LowGrayLevelZoneEmphasis | glszm | wavelet-HHL |
| ZoneEntropy | glszm | wavelet-HHL |
| SmallAreaLowGrayLevelEmphasis | glszm | wavelet-HHL |
| Coarseness | ngtdm | wavelet-HHL |
| Complexity | ngtdm | wavelet-HHL |
| Strength | ngtdm | wavelet-HHL |
| Contrast | ngtdm | wavelet-HHL |
| Busyness | ngtdm | wavelet-HHL |
| InterquartileRange | firstorder | wavelet-LLL |
| Skewness | firstorder | wavelet-LLL |
| Uniformity | firstorder | wavelet-LLL |
| Median | firstorder | wavelet-LLL |
| Energy | firstorder | wavelet-LLL |
| RobustMeanAbsoluteDeviation | firstorder | wavelet-LLL |
| MeanAbsoluteDeviation | firstorder | wavelet-LLL |
| TotalEnergy | firstorder | wavelet-LLL |
| Maximum | firstorder | wavelet-LLL |
| RootMeanSquared | firstorder | wavelet-LLL |
| 90Percentile | firstorder | wavelet-LLL |
| Minimum | firstorder | wavelet-LLL |
| Entropy | firstorder | wavelet-LLL |
| Range | firstorder | wavelet-LLL |
| Variance | firstorder | wavelet-LLL |
| 10Percentile | firstorder | wavelet-LLL |
| Kurtosis | firstorder | wavelet-LLL |
| Mean | firstorder | wavelet-LLL |
| JointAverage | glcm | wavelet-LLL |
| SumAverage | glcm | wavelet-LLL |
| JointEntropy | glcm | wavelet-LLL |
| ClusterShade | glcm | wavelet-LLL |
| MaximumProbability | glcm | wavelet-LLL |
| Idmn | glcm | wavelet-LLL |
| JointEnergy | glcm | wavelet-LLL |
| Contrast | glcm | wavelet-LLL |
| DifferenceEntropy | glcm | wavelet-LLL |
| InverseVariance | glcm | wavelet-LLL |
| DifferenceVariance | glcm | wavelet-LLL |
| Idn | glcm | wavelet-LLL |
| Idm | glcm | wavelet-LLL |
| Correlation | glcm | wavelet-LLL |
| Autocorrelation | glcm | wavelet-LLL |
| SumEntropy | glcm | wavelet-LLL |
| MCC | glcm | wavelet-LLL |
| SumSquares | glcm | wavelet-LLL |
| ClusterProminence | glcm | wavelet-LLL |
| Imc2 | glcm | wavelet-LLL |
| Imc1 | glcm | wavelet-LLL |
| DifferenceAverage | glcm | wavelet-LLL |
| Id | glcm | wavelet-LLL |
| ClusterTendency | glcm | wavelet-LLL |
| GrayLevelVariance | gldm | wavelet-LLL |
| HighGrayLevelEmphasis | gldm | wavelet-LLL |
| DependenceEntropy | gldm | wavelet-LLL |
| DependenceNonUniformity | gldm | wavelet-LLL |
| GrayLevelNonUniformity | gldm | wavelet-LLL |
| SmallDependenceEmphasis | gldm | wavelet-LLL |
| SmallDependenceHighGrayLevelEmphasis | gldm | wavelet-LLL |
| DependenceNonUniformityNormalized | gldm | wavelet-LLL |
| LargeDependenceEmphasis | gldm | wavelet-LLL |
| LargeDependenceLowGrayLevelEmphasis | gldm | wavelet-LLL |
| DependenceVariance | gldm | wavelet-LLL |
| LargeDependenceHighGrayLevelEmphasis | gldm | wavelet-LLL |
| SmallDependenceLowGrayLevelEmphasis | gldm | wavelet-LLL |
| LowGrayLevelEmphasis | gldm | wavelet-LLL |
| ShortRunLowGrayLevelEmphasis | glrlm | wavelet-LLL |
| GrayLevelVariance | glrlm | wavelet-LLL |
| LowGrayLevelRunEmphasis | glrlm | wavelet-LLL |
| GrayLevelNonUniformityNormalized | glrlm | wavelet-LLL |
| RunVariance | glrlm | wavelet-LLL |
| GrayLevelNonUniformity | glrlm | wavelet-LLL |
| LongRunEmphasis | glrlm | wavelet-LLL |
| ShortRunHighGrayLevelEmphasis | glrlm | wavelet-LLL |
| RunLengthNonUniformity | glrlm | wavelet-LLL |
| ShortRunEmphasis | glrlm | wavelet-LLL |
| LongRunHighGrayLevelEmphasis | glrlm | wavelet-LLL |
| RunPercentage | glrlm | wavelet-LLL |
| LongRunLowGrayLevelEmphasis | glrlm | wavelet-LLL |
| RunEntropy | glrlm | wavelet-LLL |
| HighGrayLevelRunEmphasis | glrlm | wavelet-LLL |
| RunLengthNonUniformityNormalized | glrlm | wavelet-LLL |
| GrayLevelVariance | glszm | wavelet-LLL |
| ZoneVariance | glszm | wavelet-LLL |
| GrayLevelNonUniformityNormalized | glszm | wavelet-LLL |
| SizeZoneNonUniformityNormalized | glszm | wavelet-LLL |
| SizeZoneNonUniformity | glszm | wavelet-LLL |
| GrayLevelNonUniformity | glszm | wavelet-LLL |
| LargeAreaEmphasis | glszm | wavelet-LLL |
| SmallAreaHighGrayLevelEmphasis | glszm | wavelet-LLL |
| ZonePercentage | glszm | wavelet-LLL |
| LargeAreaLowGrayLevelEmphasis | glszm | wavelet-LLL |
| LargeAreaHighGrayLevelEmphasis | glszm | wavelet-LLL |
| HighGrayLevelZoneEmphasis | glszm | wavelet-LLL |
| SmallAreaEmphasis | glszm | wavelet-LLL |
| LowGrayLevelZoneEmphasis | glszm | wavelet-LLL |
| ZoneEntropy | glszm | wavelet-LLL |
| SmallAreaLowGrayLevelEmphasis | glszm | wavelet-LLL |
| Coarseness | ngtdm | wavelet-LLL |
| Complexity | ngtdm | wavelet-LLL |
| Strength | ngtdm | wavelet-LLL |
| Contrast | ngtdm | wavelet-LLL |
| Busyness | ngtdm | wavelet-LLL |
