# Supplementary material for: Building nonenhanced CT based radiomics model in discriminating arteriovenous malformation related hematomas from hypertensive intracerebral hematomas
Source: Front Neurosci. 2023 Nov 28;17:1284560. doi: 10.3389/fnins.2023.1284560 (PMC10713806; doi:10.3389/fnins.2023.1284560)
Supplement: Supplementary file 2 [file Table_2.DOCX]

**Table S2. Description of the selected radiomic features with their associated feature group and filter**

| **Radiomic feature** | **Radiomic class** | **Filter** |
| --- | --- | --- |
| Maximum3DDiameter | shape | original |
| Elongation | shape | original |
| Flatness | shape | original |
| LargeDependenceLowGrayLevelEmphasis | gldm | original |
| RunLengthNonUniformity | glrlm | original |
| LargeDependenceLowGrayLevelEmphasis | gldm | logarithm |
| RunLengthNonUniformity | glrlm | logarithm |
| Median | firstorder | exponential |
| SizeZoneNonUniformity | glszm | exponential |
| SizeZoneNonUniformity | glszm | gradient |
| SizeZoneNonUniformity | glszm | square |
| LargeDependenceLowGrayLevelEmphasis | gldm | squareroot |
| RunLengthNonUniformity | glrlm | squareroot |
| SizeZoneNonUniformity | glszm | lbp-2D |
| Median | firstorder | lbp-3D-m1 |
| SizeZoneNonUniformity | glszm | lbp-3D-m1 |
| 90Percentile | firstorder | lbp-3D-m2 |
| Variance | firstorder | lbp-3D-m2 |
| SizeZoneNonUniformity | glszm | lbp-3D-m2 |
| Imc2 | glcm | lbp-3D-k |
| SizeZoneNonUniformityNormalized | glszm | lbp-3D-k |
| Variance | firstorder | wavelet-LHL |
| GrayLevelNonUniformity | glszm | wavelet-LHL |
| TotalEnergy | firstorder | wavelet-LHH |
| GrayLevelVariance | glrlm | wavelet-LHH |
| GrayLevelNonUniformityNormalized | glrlm | wavelet-LHH |
| RunVariance | glrlm | wavelet-LHH |
| GrayLevelVariance | glszm | wavelet-LHH |
| GrayLevelNonUniformityNormalized | glszm | wavelet-LHH |
| ZoneEntropy | glszm | wavelet-LHH |
| Complexity | ngtdm | wavelet-LHH |
| InterquartileRange | firstorder | wavelet-HLL |
| MaximumProbability | glcm | wavelet-HLL |
| HighGrayLevelEmphasis | gldm | wavelet-HLL |
| LowGrayLevelEmphasis | gldm | wavelet-HLL |
| GrayLevelVariance | glszm | wavelet-HLL |
| LargeAreaHighGrayLevelEmphasis | glszm | wavelet-HLL |
| ClusterShade | glcm | wavelet-LLH |
| Imc1 | glcm | wavelet-LLH |
| LargeDependenceLowGrayLevelEmphasis | gldm | wavelet-LLH |
| GrayLevelVariance | glrlm | wavelet-LLH |
| RunVariance | glrlm | wavelet-LLH |
| RunEntropy | glrlm | wavelet-LLH |
| GrayLevelVariance | glszm | wavelet-LLH |
| GrayLevelNonUniformityNormalized | glszm | wavelet-LLH |
| Skewness | firstorder | wavelet-HLH |
| SumSquares | glcm | wavelet-HLH |
| RunVariance | glrlm | wavelet-HLH |
| SmallAreaLowGrayLevelEmphasis | glszm | wavelet-HLH |
| Variance | firstorder | wavelet-HHH |
| Mean | firstorder | wavelet-HHH |
| JointEnergy | glcm | wavelet-HHH |
| DependenceNonUniformityNormalized | gldm | wavelet-HHH |
| DependenceVariance | gldm | wavelet-HHH |
| GrayLevelVariance | glrlm | wavelet-HHH |
| GrayLevelNonUniformity | glszm | wavelet-HHH |
| SmallAreaHighGrayLevelEmphasis | glszm | wavelet-HHH |
| HighGrayLevelZoneEmphasis | glszm | wavelet-HHH |
| LowGrayLevelZoneEmphasis | glszm | wavelet-HHH |
| ZoneEntropy | glszm | wavelet-HHH |
| Autocorrelation | glcm | wavelet-HHL |
| LowGrayLevelRunEmphasis | glrlm | wavelet-HHL |
| HighGrayLevelRunEmphasis | glrlm | wavelet-HHL |
| HighGrayLevelZoneEmphasis | glszm | wavelet-HHL |
| SmallAreaEmphasis | glszm | wavelet-HHL |
| LowGrayLevelZoneEmphasis | glszm | wavelet-HHL |
| Strength | ngtdm | wavelet-LLL |

Label: GLCM=Gray-level Co-occurrence Matrix,GLSZM=Gray-Level Size Zone Matrix,GLRLM=Gray Level Run Length Matrix,GLDM=Gray Level Dependence Matrix,NGTDM=Neighbouring Gray Tone Difference Matrix
